# Supplementary material for: Acute Stress Effects over Time on the Gene Expression and Neurotransmitter Patterns in the Carp (Cyprinus carpio) Brain
Source: Animals (Basel). 2024 Nov 26;14(23):3413. doi: 10.3390/ani14233413 (PMC11639763; doi:10.3390/ani14233413)

Supplementary Figure S1.

1A : Feed Reward

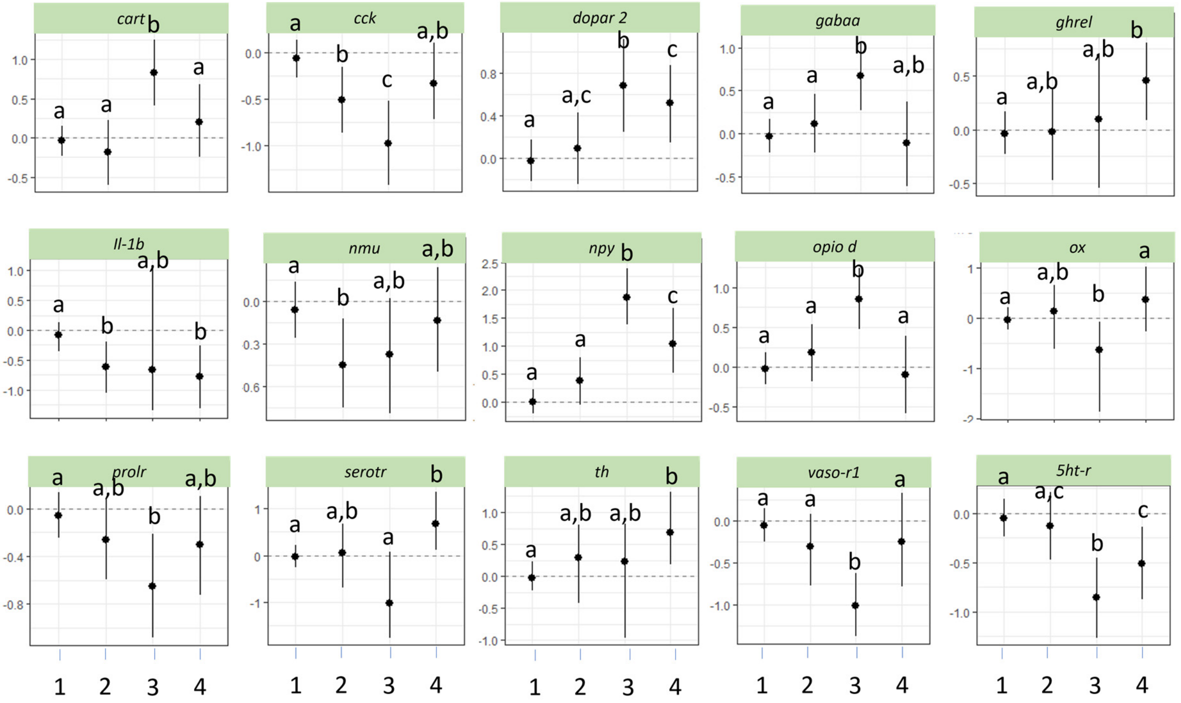

1B : Feed Contr

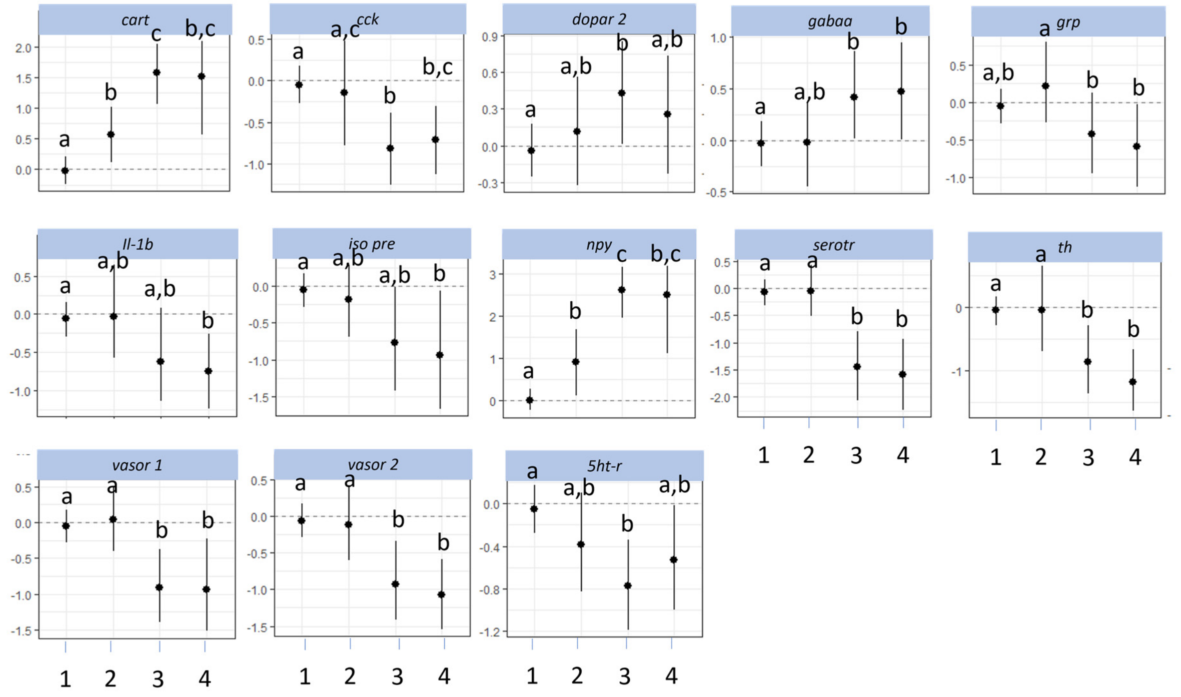

1C: Chasing

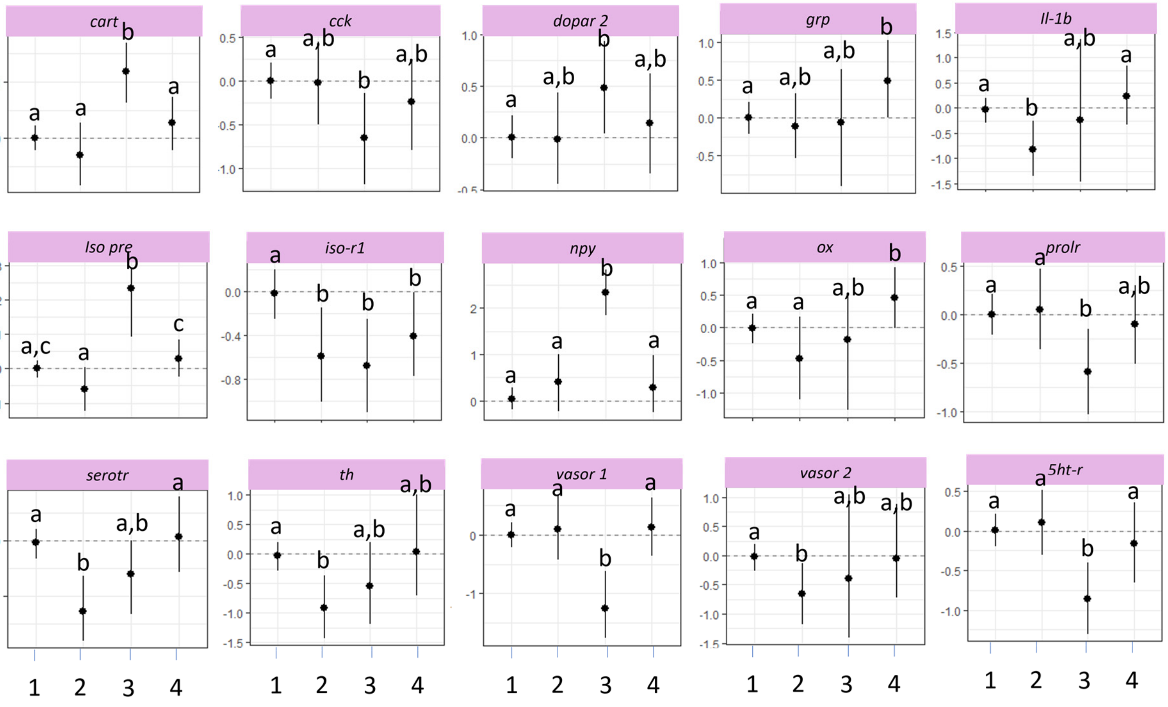

1D : Confinement

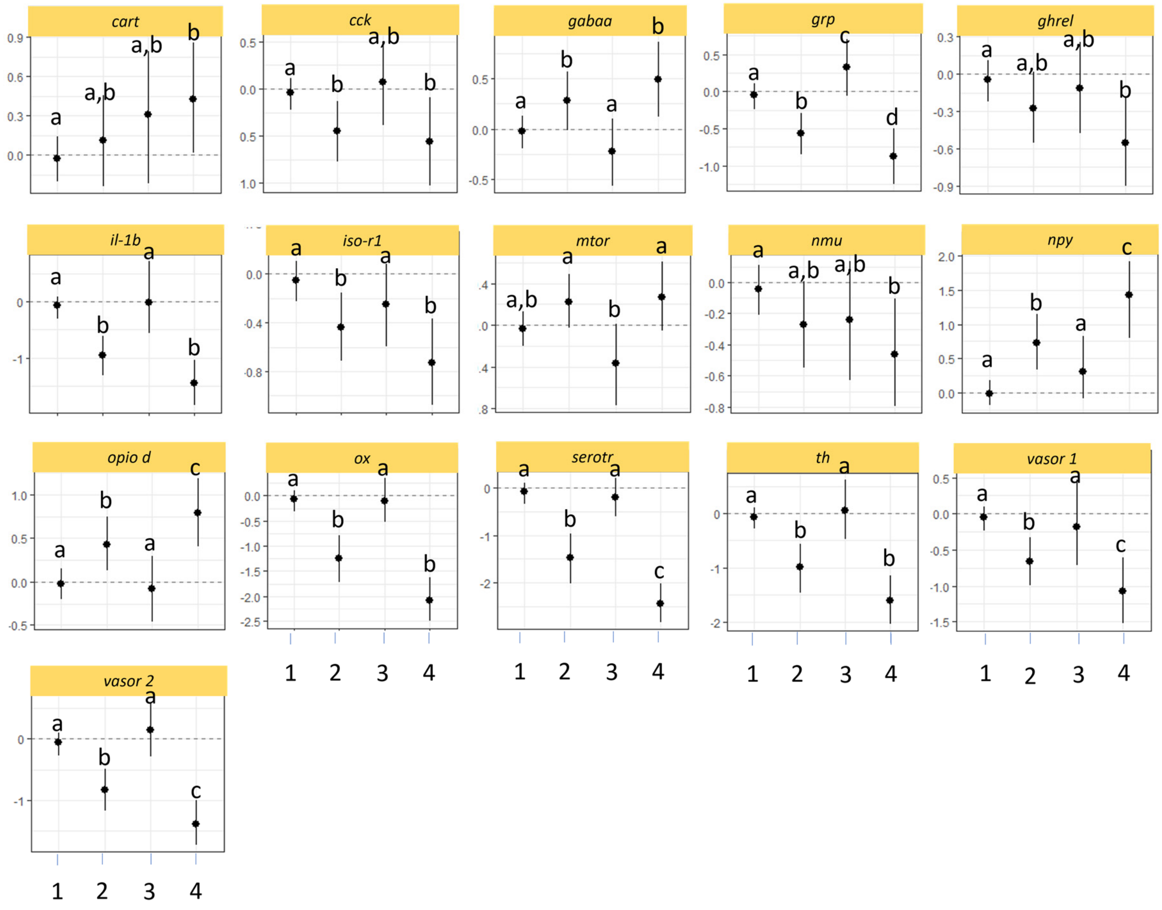

1A: Air

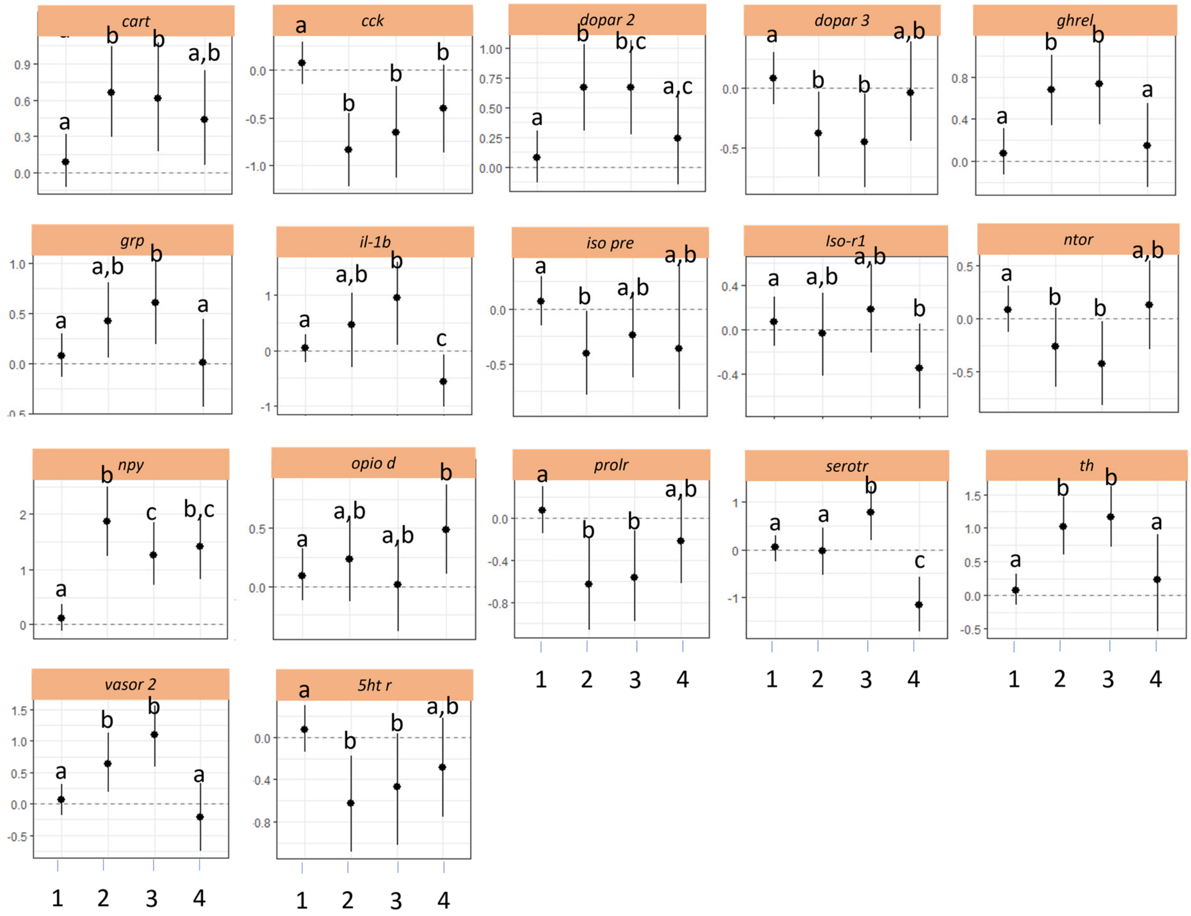

Figure S1. Gene expression profile in the telencephalon of control fish (group 1) and fish after the treatment (30 min = 2; 60 min = 3; 90 min = 4) with the different treatments as described in the Material & Methods section: feed rewarding, feed control, chasing, confinement and air exposure, marginal means ± SEM; n = 6 per treatment, means of groups with the same letters are not significantly different from each other, p > 0.05).

For feed-rewarded carp, the expression of *nmu* was significantly lower 30 min after treatment compared with the controls (p = 0.008; Fig. S1A), and the expression of *ghrel* higher 90 min after treatment compared with the controls (p = 0.014). In the same fish, the *cart* expression was found to be significantly higher in the 60 min group compared with the remaining treatment groups (p ≤ 0.042). In addition, a significantly higher *npy* expression was observed in the 60 min and the 90 min groups compared with the controls and fish sampled after 30 min (p ≤ 0.032). Furthermore, the expression of *cck* was significantly lower in the 30 min and 60 min groups compared with the controls (p ≤ 0.004), and significantly higher 90 min after feed rewarding compared with the fish sampled after 60 min (p = 0.028). In parallel, the ox expression was lower 60 min after treatment compared with the controls and the 90 min group (p ≤ 0.040). For the feed-rewarded carp, the expression of *5ht-r* was significantly lower in the 60 min and 90 min group than in the controls (p ≤ 0.016; Fig. S1A) and lower 60 min after treatment compared with the fish sampled 30 min after feed rewarding (p = 0.004). Moreover, there was a significantly higher *serotr* expression in the 90 min group than in the controls or the 30 min group (p ≤ 0.012). Finally, the *dopar2* expression in feed-rewarded animals was significantly lower in the 60 min and 90 min groups than in the controls (p ≤ 0.006), and also significantly different between fish sampled after 30 min and 60 min after treatment (p = 0.022). In addition, the expression of *gabaa* was significantly higher 60 min after feed rewarding compared with the controls and the fish samples after 30 min (p ≤ 0.024). In the same fish, also the expression of the *vaso-r1* was found to be lower in fish 60 min (p = 0.022) and the expression of *opiod* higher 60 min after feed rewarding compared with the remaining treatment groups (p ≤ 0.006). Fish belonging to the feed reward group showed lower *prolr* expression in the 60 min group than in the controls (p ≤ 0.012). The expression of *th* was higher 90 min after treatment compared with the controls (p = 0.006). Finally, the expression of *il-1β* was lower

30 min and 90 min after feed rewarding compared with the controls ($p \leq 0.016$). The expression of *cart* and *npy* in the feed control animals was significantly higher in all sham-treated fish compared with the controls ($p \leq 0.024$; Fig. S1B). In addition, feed control fish showed lower *grp* expression 60 min and 90 min after treatment compared with the fish sampled after 30 min ($p \leq 0.030$). Moreover, the *cck* expression was found to be lower 60 min and 90 min after treatment compared with the controls ($p \leq 0.004$), and the *cck* expression was also lower 60 min after treatment compared with the fish sampled 30 min after treatment ($p = 0.028$). For feed control fish, the expression of *5ht-r* was significantly lower in fish 60 min after the sham treatment compared with the controls ($p < 0.001$; Fig. S1B). The expression of *serotr* in the feed control groups was lower in the groups 60 min and 90 min after the treatment compared with the controls ($p < 0.001$), and also the expression of *iso pre* was found to be lower and the expression of *gabaa* in these fish higher 90 min after the treatment compared with the controls ($p \leq 0.046$). Moreover, the *il-1β* expression was lower 90 min after treatment compared with the controls ($p = 0.044$). The expression of *vasor 1, vasor 2* and *th* was significantly lower 60 min and 90 min after treatment compared with the remaining two treatment groups ($p \leq 0.026$). The expression of *dopar 2* was found to be higher in animals 60 min after the treatment compared with the controls ($p = 0.030$). Chasing resulted in higher *cart* expression compared with the remaining treatments ($p \leq 0.008$; Fig. S1C). In addition, chased fish showed significantly higher *npy* expression in fish 60 min after treatment compared with the remaining treatments ($p \leq 0.001$). The expression of *cck* was lower 60 min after treatment than in the controls ($p = 0.008$). In addition, a higher *grp* and *ox* expression was observed in the 90 min compared with the controls ($p = 0.038$), whereby the *ox* expression was also higher in the 90 min compared with the 30 min group ($p = 0.020$). Chasing resulted in lower expression of *5ht-r* and *vasor 1* in fish 60 min after treatment compared with the remaining treatments ($p \leq 0.024$; Fig. S1C). In addition, chased fish showed lower *serotr* expression 30 min compared with the controls and fish sampled after 90 min ($p \leq 0.004$). The expression of *iso pre* was lower in fish sampled after 30 min compared with the fish sampled after 60 min and 90 min ($p \leq 0.038$). The expression of *dopar 2* was higher in fish sampled after 60 min than in the controls ($p = 0.032$). In contrast, the expression of *prolr* was lower 60 min after treatment compared with the controls and fish sampled after 30 min ($p \leq 0.022$). The expression of *th* and *vasor 2* showed lower expressions in fish 30 min after chasing compared with the controls ($p \leq 0.012$). Additionally, the expression of the *it-r1* was lower in all chased fish compared with the controls ($p \leq 0.042$). Finally chasing resulted in lower *il-1b* expression 30 min after treatment compared with the controls and fish sampled 90 min after chasing ($p \leq 0.022$). Confinement resulted in a significantly lower *nmu* expression 90 min after treatment compared with the controls ($p = 0.032$; Fig. S1D). Furthermore, the expression of *cart* was significantly higher in the 90 min group compared with the controls ($p = 0.046$). Confinement also resulted in higher *npy* expression and lower *cck* and *ox* expression 30 min and 90 min after treatment compared with the controls ($p \leq 0.042$), whereby the *ox* expression was also different between the 60 min group and the other two confined groups ($p \leq 0.001$). The expression of *ghrel* was lower in the 90 min group than in the controls ($p = 0.012$). In addition, confinement resulted in a different *grp* expression in all treatment groups ($p \leq 0.042$). Moreover, confinement led to lower *serotr* expression in fish 30 min and 90 min after treatment compared with the controls and fish after 60 min ($p \leq 0.001$; Fig. S1D). Confined animals also showed significantly higher *mtor* expression in the controls and in the 90 min group compared with the 60 min group ($p \leq 0.012$). Finally, confinement resulted in significantly higher *gabaa* and *opiod* expression in fish 30 min and 90 min after treatment compared with the controls and fish of the 60 min group ($p \leq 0.032$). Confined fish also showed lower *vasor 1, vasor 2, it-r1, il-1β* and *th* expression 30 min and 90 min after treatment compared with the controls or the 60 min group ($p \leq 0.036$). Air exposure resulted in higher *cart* and *ghrel* expression 30 min and 60 min after treatment compared with the controls ($p \leq 0.020$; Fig. S1E). Furthermore, the expression of *npy* was higher in all air-exposed fish than in the controls ($p \leq 0.001$). Moreover, the expression of *grp* was higher 60 min after air exposure compared with the controls and fish sampled after 90 min ($p \leq 0.048$). Moreover, air-exposed fish showed lower *cck* expression than the controls (≤ 0.034). Air exposure influenced the *5ht-r* expression which was lower in the fish 30 min and 60 min after treatment compared with the controls ($p \leq 0.036$; Fig. S1E). In addition, the expression of *serotr* was higher 60 min and lower 90 min after treatment compared with the controls and fish sampled after 30 min ($p \leq 0.012$). In the same fish, the expression of *iso pre* was lower 30 min after treatment than in the controls ($p = 0.016$). In addition, the expression of *mtor* and *dopar 3* was lower and the expression of *dopar 2* and *prolr* higher 30 min and 60 min after treatment compared with the controls ($p \leq 0.046$). In contrast, the expression of *th* and *vasor 2* was higher 30 min and 60 min after treatment compared with the controls and fish sampled 90 min

after treatment ($p \leq 0.048$). The expression of *opiod* was higher and the expression of *it-r1* lower 90 min after treatment compared with the controls ($p = 0.028$). In addition, the *il-1β* expression was higher 60 min and lower 90 min after air exposure compared with the controls ($p \leq 0.044$).

Supplementary Figure S2.

2A : Feed reward

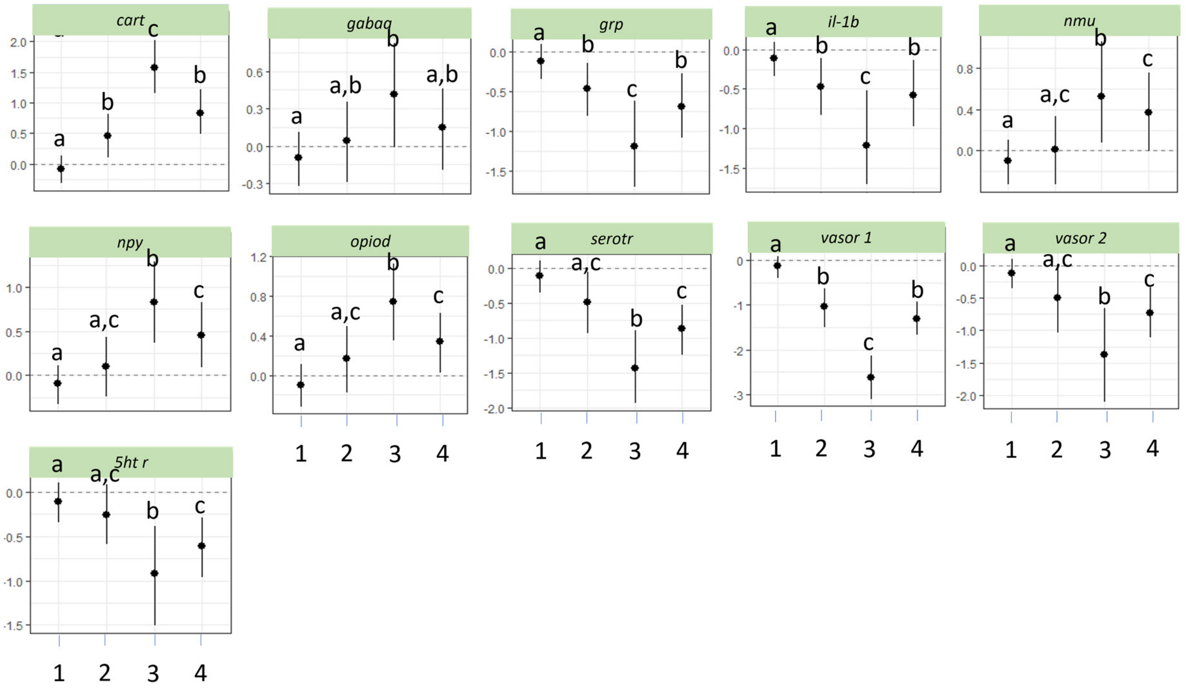

2B : Feed Contr

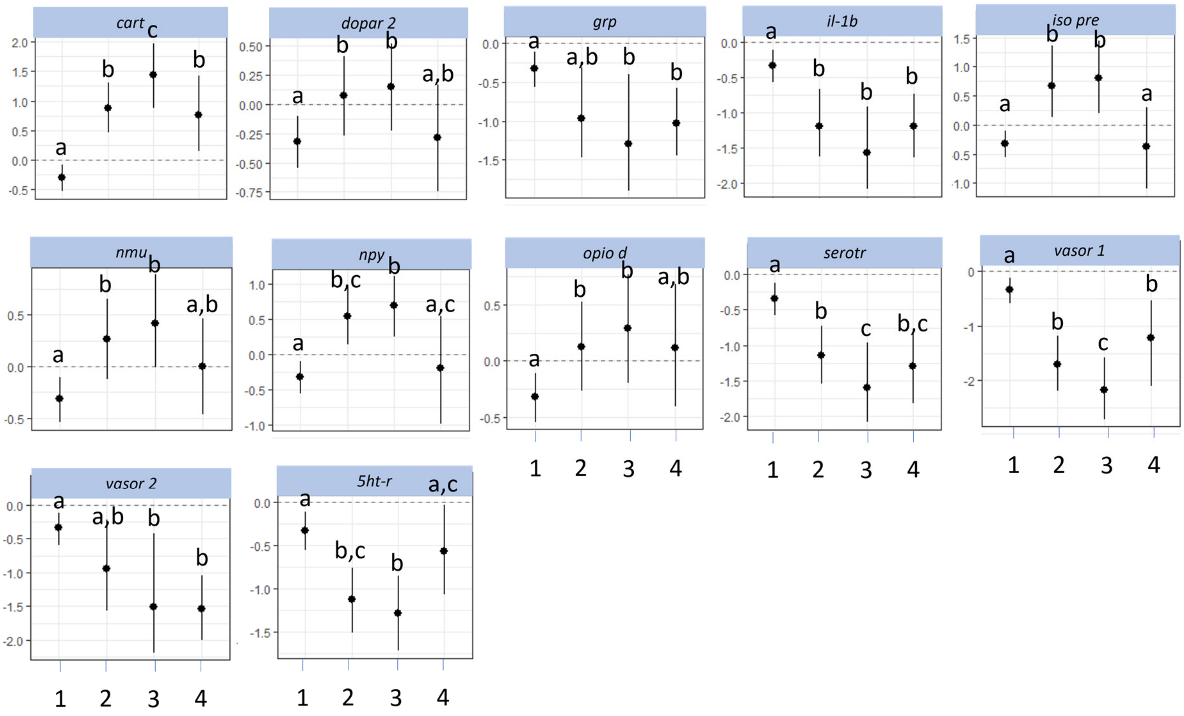

2C : Chasing

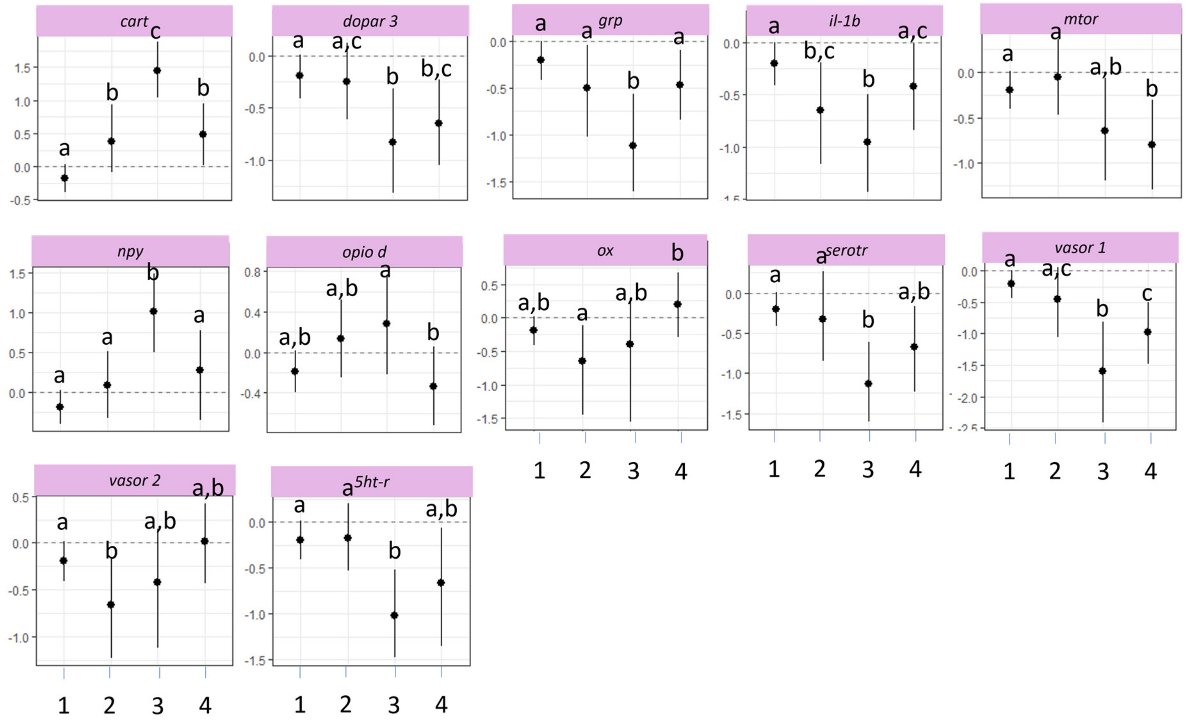

2D : Confinement

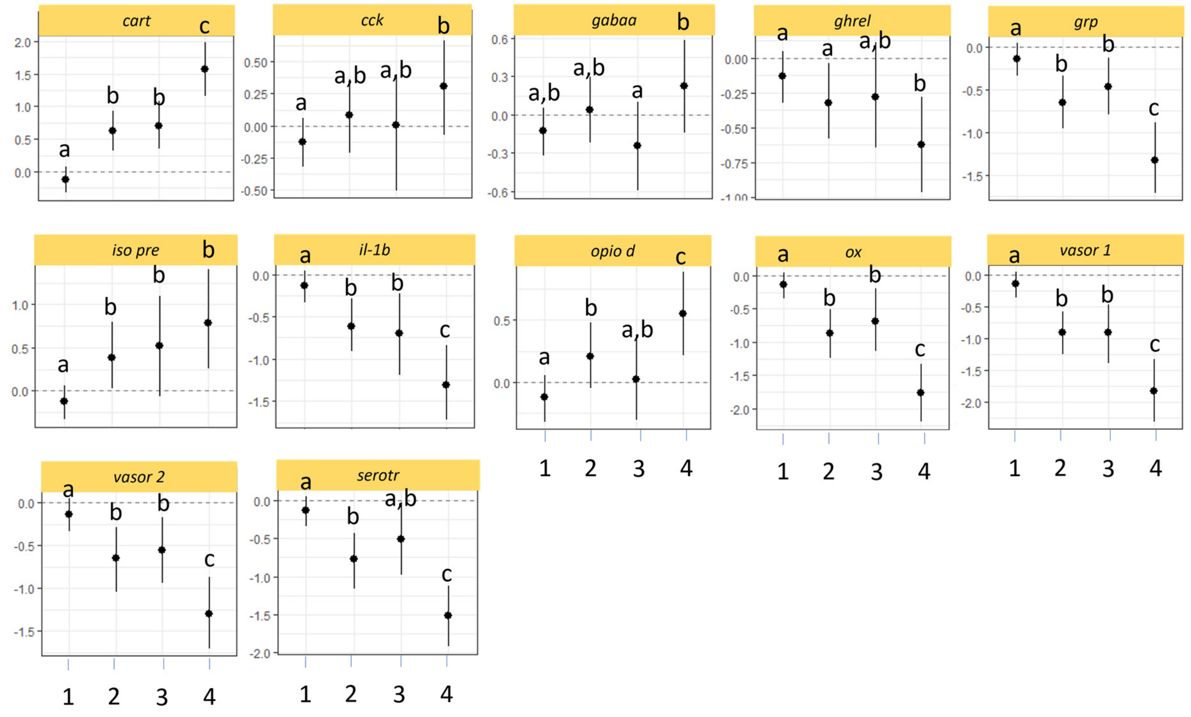

2E : Air

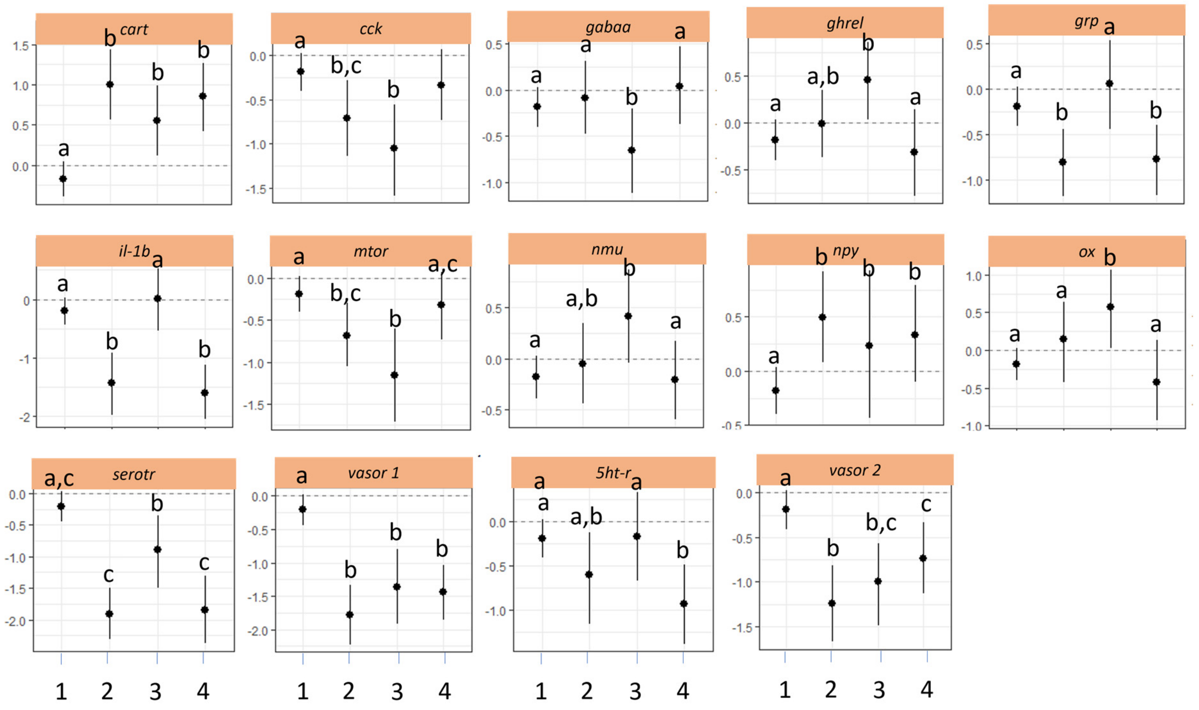

Figure S2. Gene expression profile in the hypothalamus of control fish (group 1) and fish after the treatment (30 min = 2; 60 min = 3; 90 min = 4) whereby the following different treatments have been applied as described in the Material & Methods section: feed rewarding, feed control, chasing, confinement and air exposure, marginal means ± SEM; n = 6 per treatment. means of groups with the same letters are not significantly different from each other, $p > 0.05$).

For the hypothalamus of carp, the feed rewarding had an increasing effect on the *nmu* and *npy* expression 60 min and 90 min after the treatment compared with the controls ($p \leq 0.010$; Fig. S2A). In addition, the expression of *cart* and the expression of *grp* lower in the hypothalamus was higher in all feed-rewarded fish compared with the controls ($p \leq 0.044$). For the hypothalamus of carp, the feed-rewarded groups also showed a significantly lower *5ht-r, serotr, vasor 1* and *vasor 2* expression 60 min and 90 min after the treatment compared with the controls ($p < 0.004$; Fig. S2A). In contrast, a higher *opio d* expression was observed expression 60 min and 90 min after the treatment compared with the controls ($p \leq 0.008$). Moreover, the expression of *gabaa* was significantly higher in the feed-rewarded fish 60 min after treatment compared with the controls ($p \leq 0.026$). In addition, in all feed-rewarded animals the expression of *il-1β* was lower compared with the controls ($p \leq 0.038$). In addition, the *nmu* and *npy* expression was higher in fish 30 min and 60 min after feed control treatment compared with the controls ($p \leq 0.002$; Fig. S2B). In the feed control fish, the expression of *cart* was higher in all treated fish compared with the controls ($p \leq 0.001$). Moreover, the expression of *grp* was lower in the 60 min and 90 min groups compared with the controls ($p \leq 0.038$). In the feed control fish, the expression of *5ht-r* in the hypothalamus was significantly lower and the expression of *opiod* higher in the fish 30 min and 60 min after the treatment compared with the controls ($p \leq 0.001$; Fig. 2B). In addition, all feed control fish showed lower expression of *serotr* compared with the controls ($p \leq 0.001$). The expression of *iso pre* was significantly higher 30 min and 60 min after the treatment compared with the controls and fish samples 90 min after feed control treatment ($p \leq 0.012$). In the feed control fish, the expression of *dopar 2b* in the hypothalamus was significantly higher in the fish 30 min and 60 min after the treatment compared with the controls ($p \leq 0.028$; Fig. S2B), and the expression of *vasor 1* and *il-1β* was lower in all sham-treated fish compared with the controls ($p \leq 0.012$). In addition, the expression of *vasor 2* was lower 60 min and 90 min after feed control treatment compared with the controls ($p \leq 0.034$). The expression of *cart* showed higher values in all chased fish compared with the controls ($p = 0.012$; Fig. S2C), whereas the expression of *npy* was higher and the expression of *grp* lower 60 min after chasing compared with the remaining treatments ($p \leq 0.010$). The expression of *ox* was higher 90 min

after chasing than 30 min after this treatment ($p = 0.024$). The expression of *5ht-r* and *serotr* was significantly lower 60 min after chasing compared with the controls and fish sampled after 30 min ($p \leq 0.006$; Fig. S2C). In the same treatment, the expression of *mtor* was significantly lower in fish 90 min after chasing compared with the controls and fish sampled after 30 min ($p \leq 0.022$). In the same treatment groups, the expression of the *vasor 1* and *dopar 3* was lower in the 60 min and the 90 min group compared with the controls ($p \leq 0.001$). The expression of *vasor 2* was lower 30 min after the chasing compared with the controls ($p \leq 0.032$), and the expression of *il-1β* lower 30 min and 60 min after treatment compared with the controls ($p \leq 0.036$). The fish sampled 90 min after chasing showed a lower *opio d* expression than the 60 min group ($p = 0.020$). In the hypothalamus of confined fish, the expression of *cart* was significantly higher and the expression of *ox* lower in all fish after the treatment compared with the controls ($p \leq 0.001$; Fig. S2D). In addition, the expression of *cck* was higher 90 min after confinement than in the controls ($p = 0.032$), and the expression of *ghrel* lower after 90 min compared with the controls and the fish sampled after 30 min ($p \leq 0.038$). In the hypothalamus of confined fish, the expression of *serotr* was significantly lower in the fish 30 min and 90 min after the treatment compared with the controls ($p < 0.001$; Fig. S2D). The same fish also showed higher *iso pre* expression after confinement compared than in the controls ($p \leq 0.028$). The expression of *gabaa* was significantly higher 90 min after treatment compared with the 60 min group ($p = 0.038$). Confined fish also showed a lower expression of *il-1β*, *vasor 1* and *vasor 2* compared with the controls ($p \leq 0.002$), and a lower *opiod* expression 30 min and 90 min after treatment compared with the controls ($p \leq 0.018$). In the hypothalamus of air-exposed fish, the expression of *nmu, ox* and *ghrel* was significantly higher in fish 60 min after the treatment compared with controls and fish sampled after 90 min ($p = 0.032$; Fig. S2E). In addition, the expression of *cart* was higher in all air-exposed fish compared with the controls ($p \leq 0.001$). The expression of *npy* was higher and the expression of *grp* lower 30 min and 90 min after treatment compared with the controls ($p \leq 0.022$). Quite similar, the expression of *cck* lower 30 min and 60 min after treatment compared with the controls ($p \leq 0.016$). In the hypothalamus of air-exposed fish, significantly lower expression values of *5ht-r, vasor 1* and *serotr* were observed in all fish after treatment compared with the controls ($p \leq 0.008$; Fig. S2E). The expression of *mtor* was lower 30 min and 60 min after treatment compared with the controls ($p \leq 0.014$). In contrast, the expression of *gabaa* was lower 60 min after treatment compared with all remaining treatments ($p \leq 0.042$), and the expression of *vasor 2* was lower 90 min after treatment than in the controls or in the 60 min group ($p \leq 0.024$). The expression of *il-1β* was lower 30 min and 90 min after treatment compared with the controls and fish sampled after 60 min after air exposure ($p \leq 0.001$).

Supplementary Figure S3.

3A : Feed reward

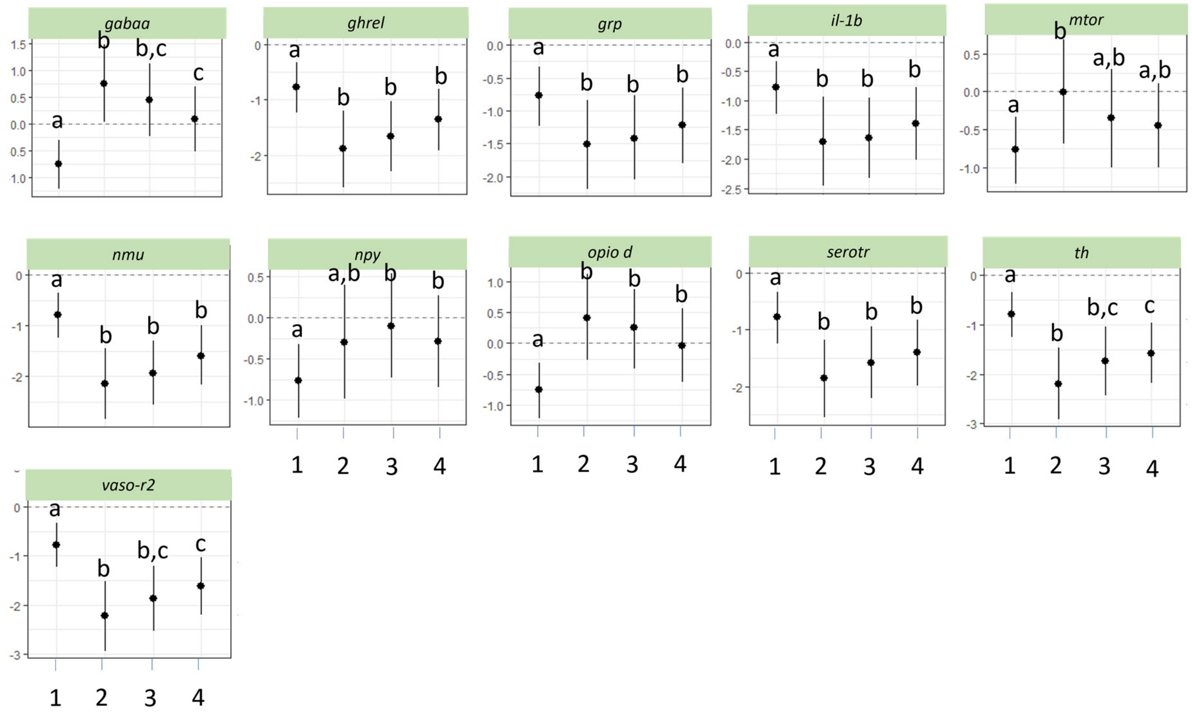

3B : Feed Contr

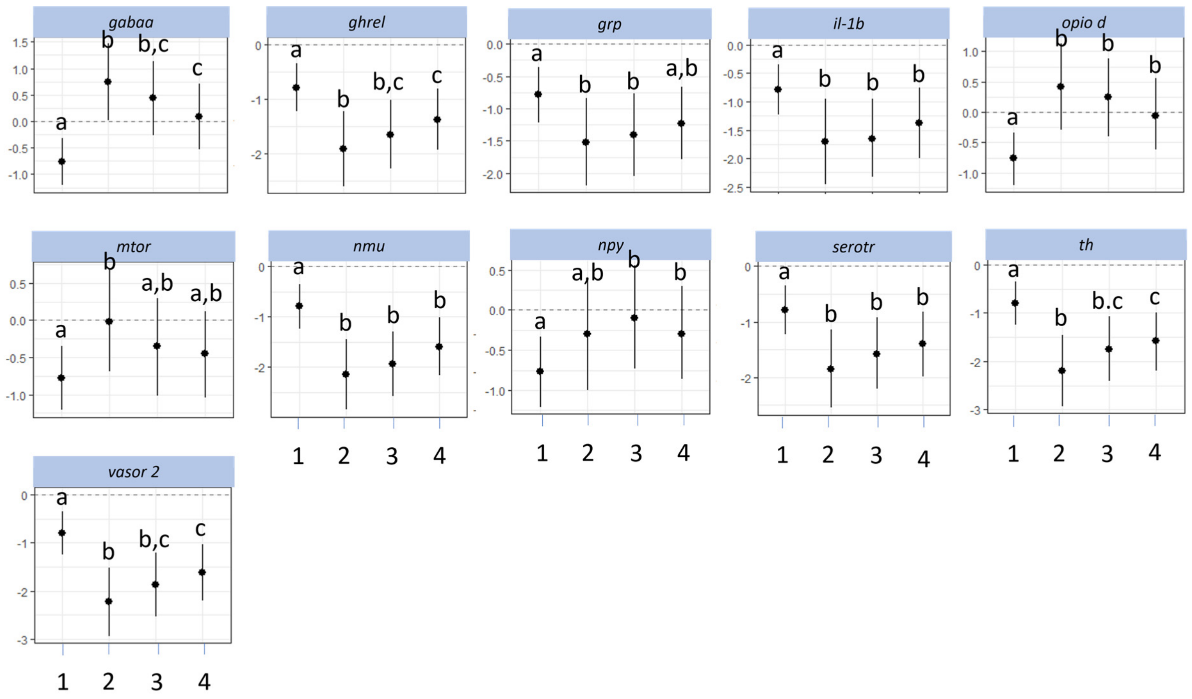

3C : Chasing

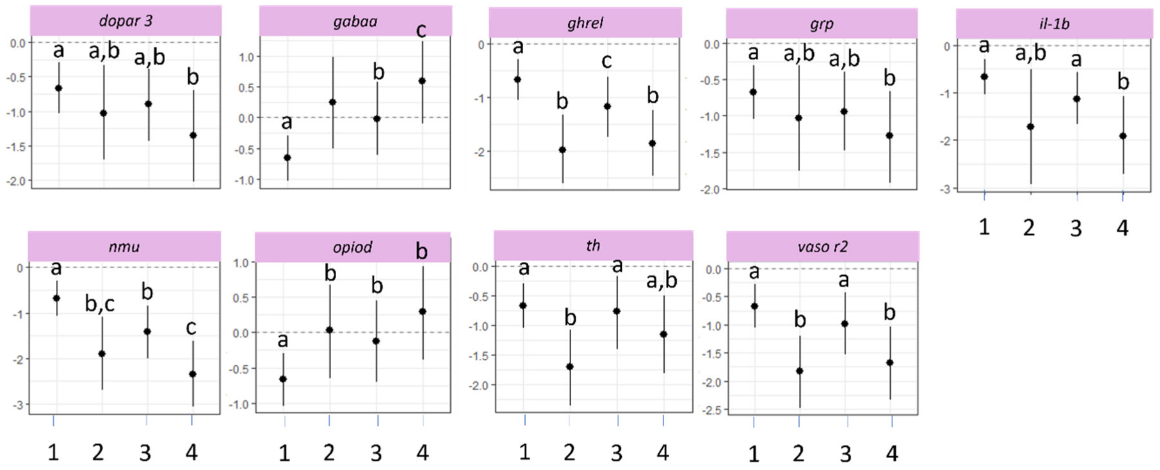

3D : Confinement

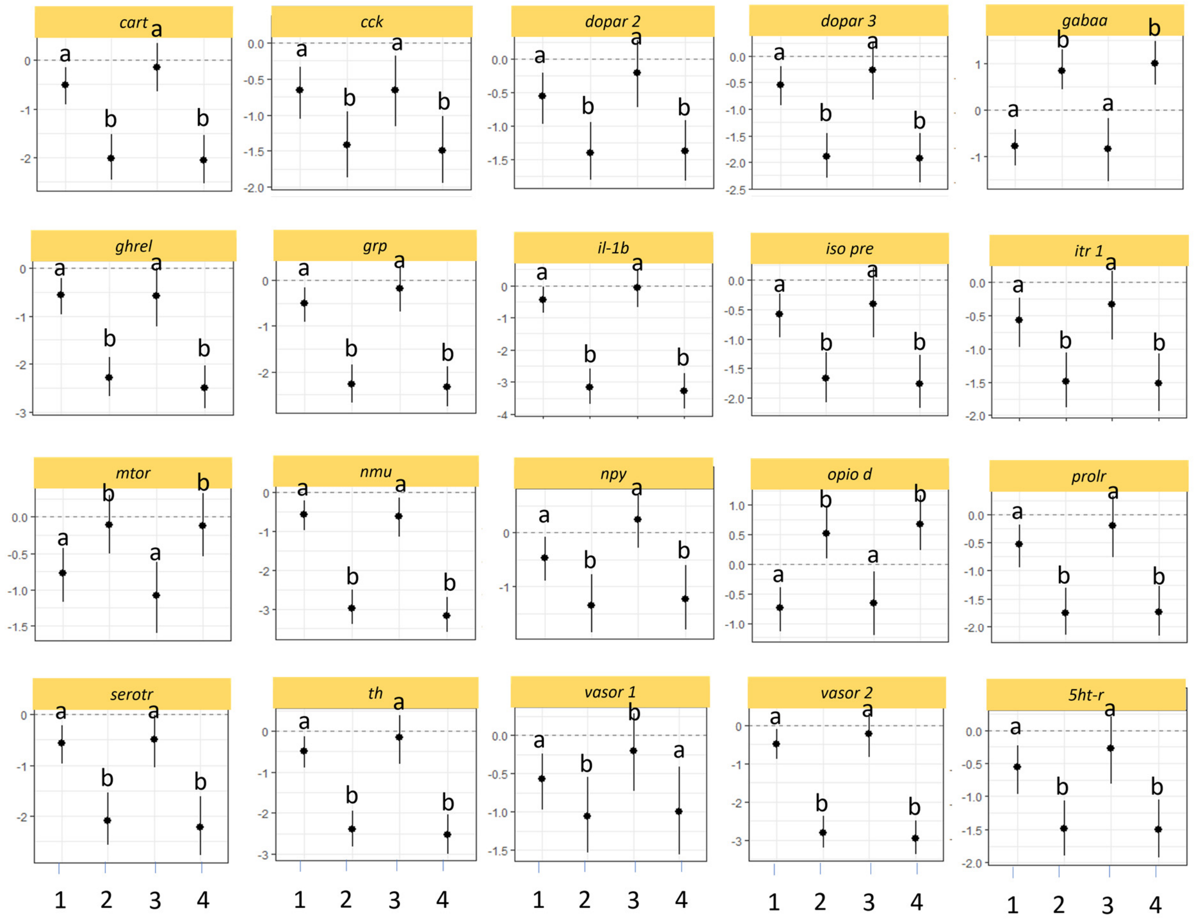

3E : Air

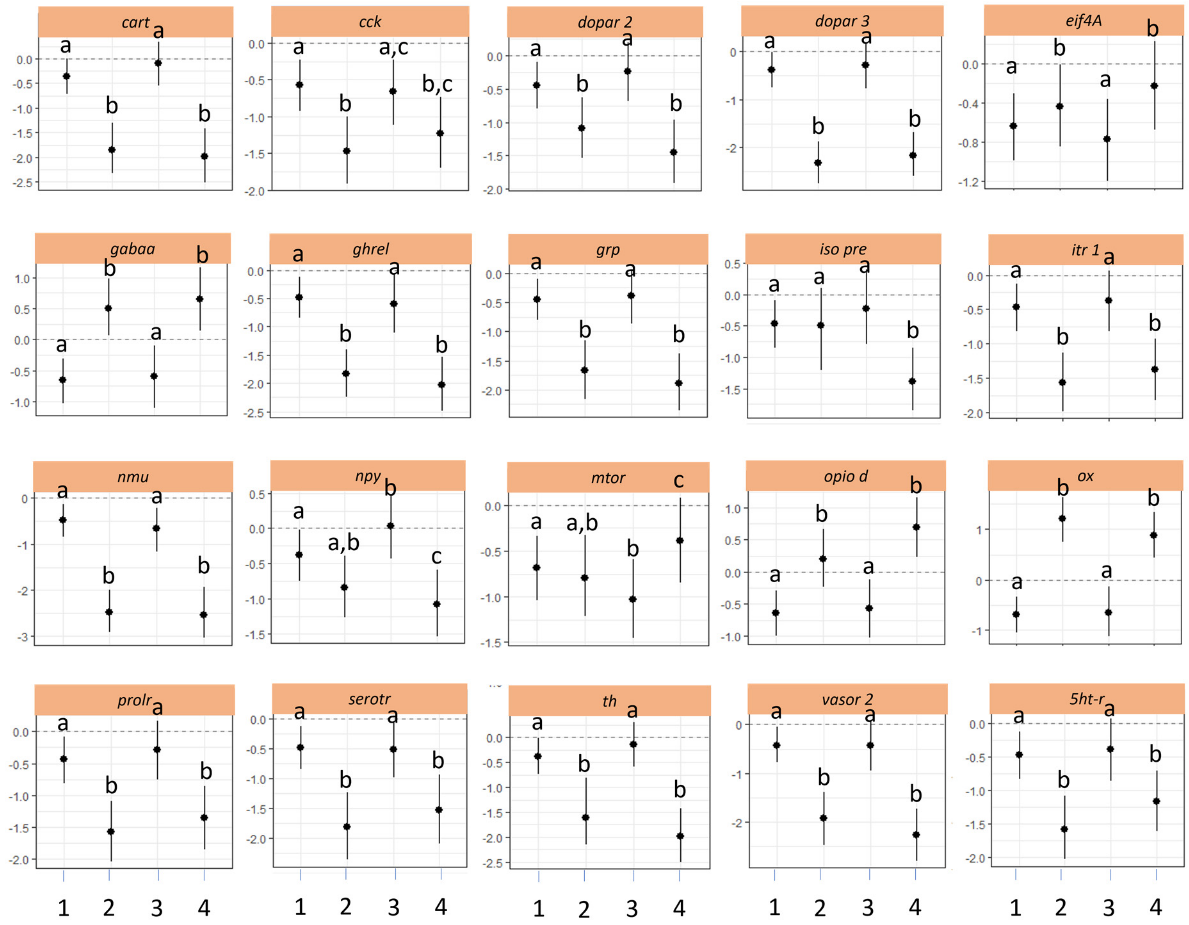

Figure S3. Gene expression profile in the optic tectum of control fish (group 1) and fish after the treatment (30 min = 2; 60 min = 3; 90 min = 4) whereby the following different treatments have been applied as described in the Material & Methods section: feed rewarding, feed control, chasing, confinement and air exposure, marginal means ± SEM; n = 6 per treatment, means of groups with the same letters are not significantly different from each other, p > 0.05).

In the optic tectum of carp, the expression of *nmu, ghrel* and *grp* was significantly lower in all fish after feed rewarding compared with the controls (p ≤ 0.046; Fig. S3A). The expression of *npy* in feed-rewarded fish was significantly higher 60 min and 90 min after treatment compared with the controls (p ≤ 0.030). In the optic tectum of carp, the expression of *serotr* was significantly lower in all feed-rewarded fish and the expression of *gabaa* higher in all feed-rewarded fish compared with controls (p ≤ 0.010; Fig. S3A). In contrast, the expression of *mtor* was significantly higher 30 min after the treatment compared with controls (p = 0.018). The expression of *th* was lower in all feed-rewarded fish compared with the controls (p ≤ 0.002), whereas the expression of *vasor* 2 and *serotr* was lower in all feed-rewarded fish compared with the controls (p ≤ 0.001). The expression of *il-1β* was lower and the expression of *opiod* was found to be higher in all feed-rewarded fish compared with the controls (p ≤ 0.012). In feed control fish, the expression of *nmu* and *ghrel* was significantly lower in all fish after treatment compared with the controls (p ≤ 0.012; Fig. S3B). The expression of *mtor* was higher 30 min after treatment than in the controls (p = 0.012). In addition, the expression of *npy* was higher in all fish 60 and 90 min after treatment compared with the controls (p ≤ 0.032). The expression of *grp* was lower 30 and 60 min after feed control treatment compared with the controls (p ≤ 0.026). Similarly, the expression of *il-1β* was lower and the expression of *opiod* was found to be higher in all feed control fish compared with the controls (p ≤ 0.014; Fig. S3B). The expression of *serotr, vasor* 2 and *th* was lower in all fish of the feed control groups than in the controls (p ≤ 0.006). In addition, the feed control fish showed higher *mtor* expression 30 min after treatment than in the controls (p = 0.012). The expression of *nmu* showed lower values in all chased fish compared with the controls (p ≤ 0.002; Fig. S3C). Moreover, the expression of *grp* was lower 90 min after treatment compared with the controls (p ≤

0.044). The expression of *ghrel* was lower 30 and 90 min after feed control treatment compared with the controls and fish sampled after 60 min ($p \leq 0.026$). The expression of *gabaa* and *opiod* in chased fish showed higher values in all chased fish after treatment compared with the controls ($p \leq 0.016$; Fig. S3C). In addition, the *dopar 3* expression after 90 min was lower than in the controls ($p \leq 0.034$). The expression of *vasor 2* was lower in the 30 min and 90 min groups than in the controls and fish sampled 60 min after treatment ($p \leq 0.016$). Finally, chased fish showed a lower *il-1β* expression 90 min after treatment compared with the controls and fish sampled 60 min after treatment ($p \leq 0.026$). In confined fish, the expression of *5ht-r, serotr, dopar 2, dopar 3, il-1β, gabaa, prolr, th, vasor 2, itr 1* and *iso pre* was significantly lower in fish sampled 30 min and 90 min after the treatment compared with the controls and fish sampled after 60 min ($p \leq 0.002$; Fig. 3D), but the expression of *mtor* and *opio d* was higher in the 30 min and 90 min group than in the 60 min group and the controls ($p \leq 0.012$). Moreover, all confined fish showed a higher *vasor 1* expression 60 min after treatment compared with remaining treatments ($p \leq 0.032$). In confined fish, the expression of *nmu, npy, ghrel, grp, cck* and *cart* was significantly higher in the controls and in fish sampled after 60 min compared with the other two treatment groups ($p \leq 0.042$; Fig. S3D). Air-exposed fish showed lower *cart, ghrel* and *grp* expression in the optic tectum at 30 min and 90 min after treatment compared with the controls and fish sampled after 60 min ($p < 0.001$; Fig. S3E). Air-exposed animals also showed higher *nmu* expression in controls and 60 min after treatment compared with the remaining two treatments ($p < 0.001$), and higher *npy* expression 60 min after treatment and lower *npy* expression 90 min after treatment compared with the controls ($p \leq 0.012$). However, the expression of *cck* was lower and the expression of *ox* higher in the 30 min and the 90 min group than in the controls ($p \leq 0.018$). Air-exposed fish showed lower *5ht-r* and *serotr, vasor 2, th, prolr, itr 1, dopar 2* and *dopar 3* expression and higher *gabaa* and *opio d* expression in the optic tectum 30 and 90 min after treatment compared with the controls ($p \leq 0.012$; Fig. S3E). In addition, the *iso pre* expression was found to be lower in the 90 min compared with the other treatments ($p \leq 0.008$). However, the expression of *mtor* was lower 60 min after treatment and higher 90 min after treatment compared the controls ($p \leq 0.040$).

Supplementary Figure S4.

4A : Feed reward

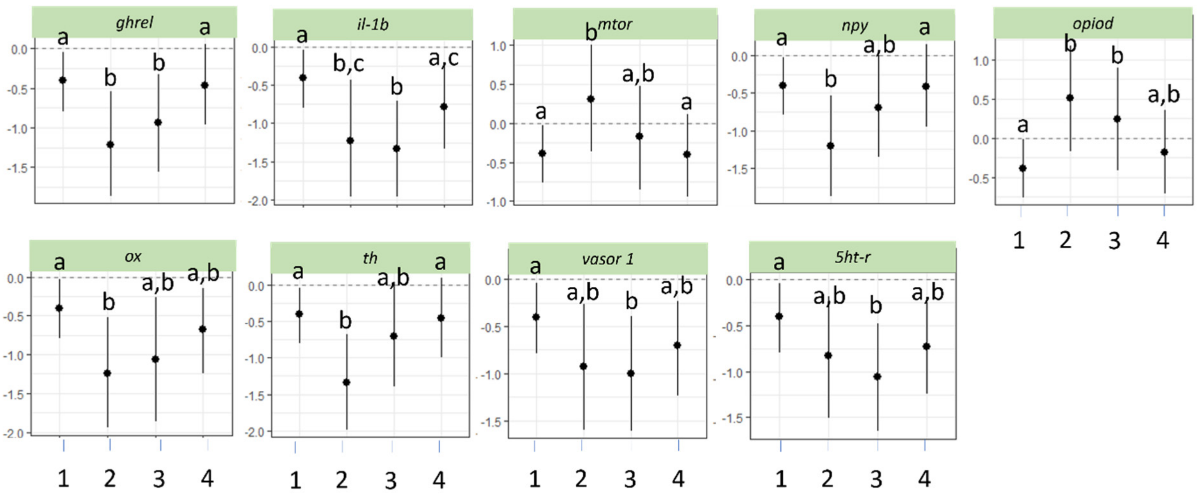

4B : Feed Contr

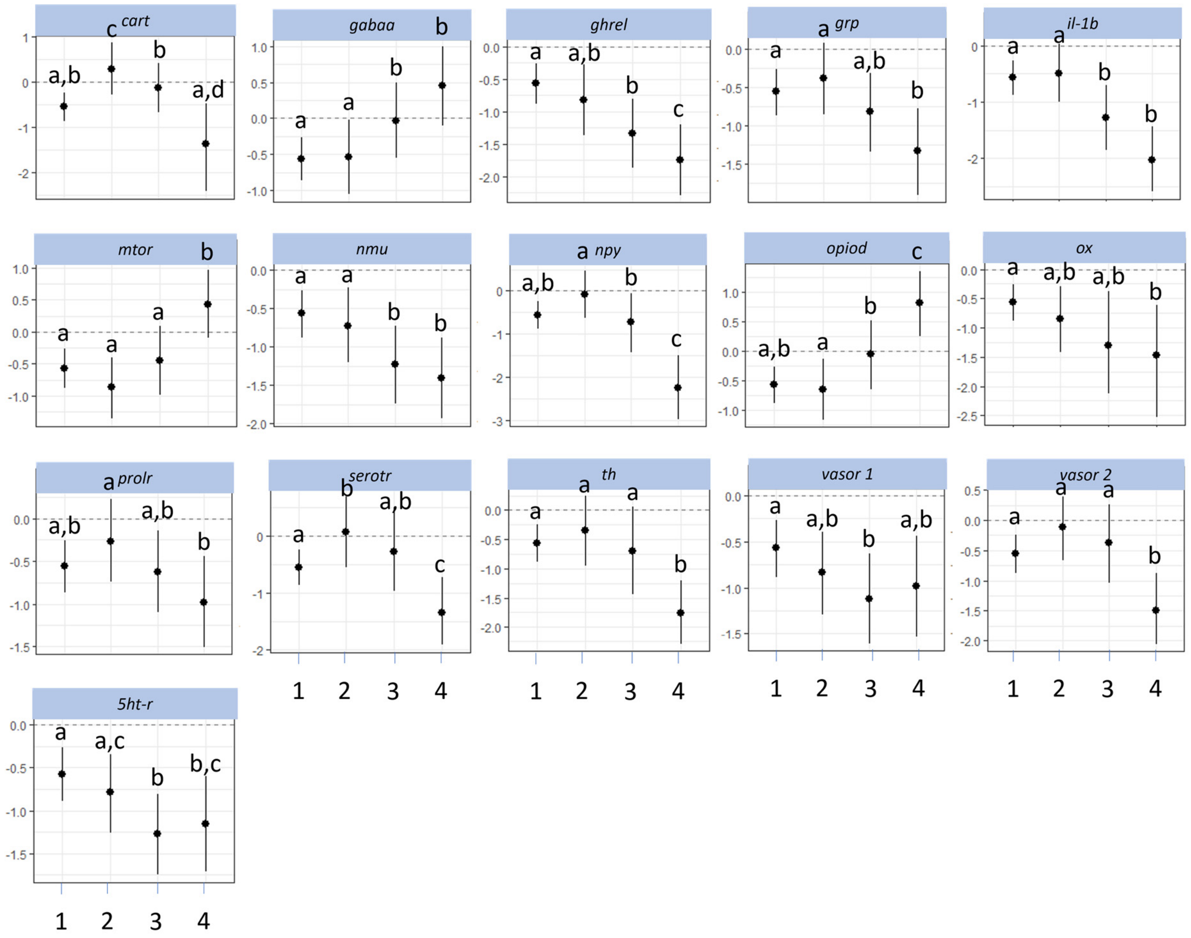

4C : Chasing

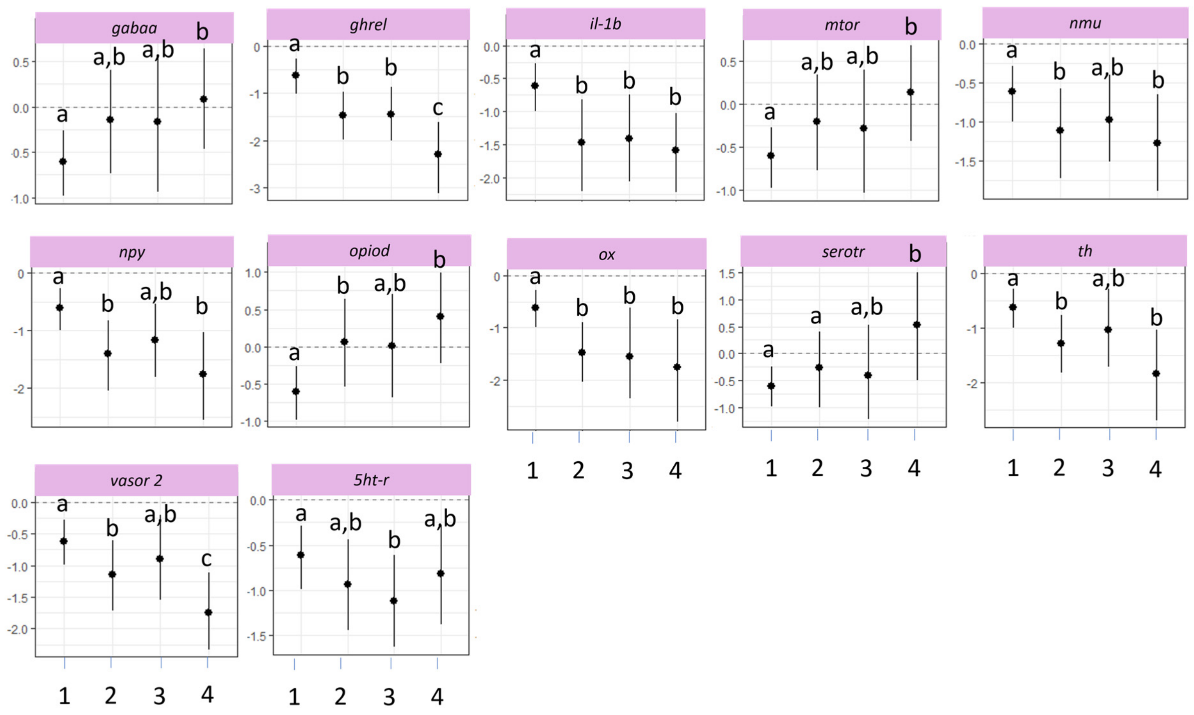

4D : Confinement

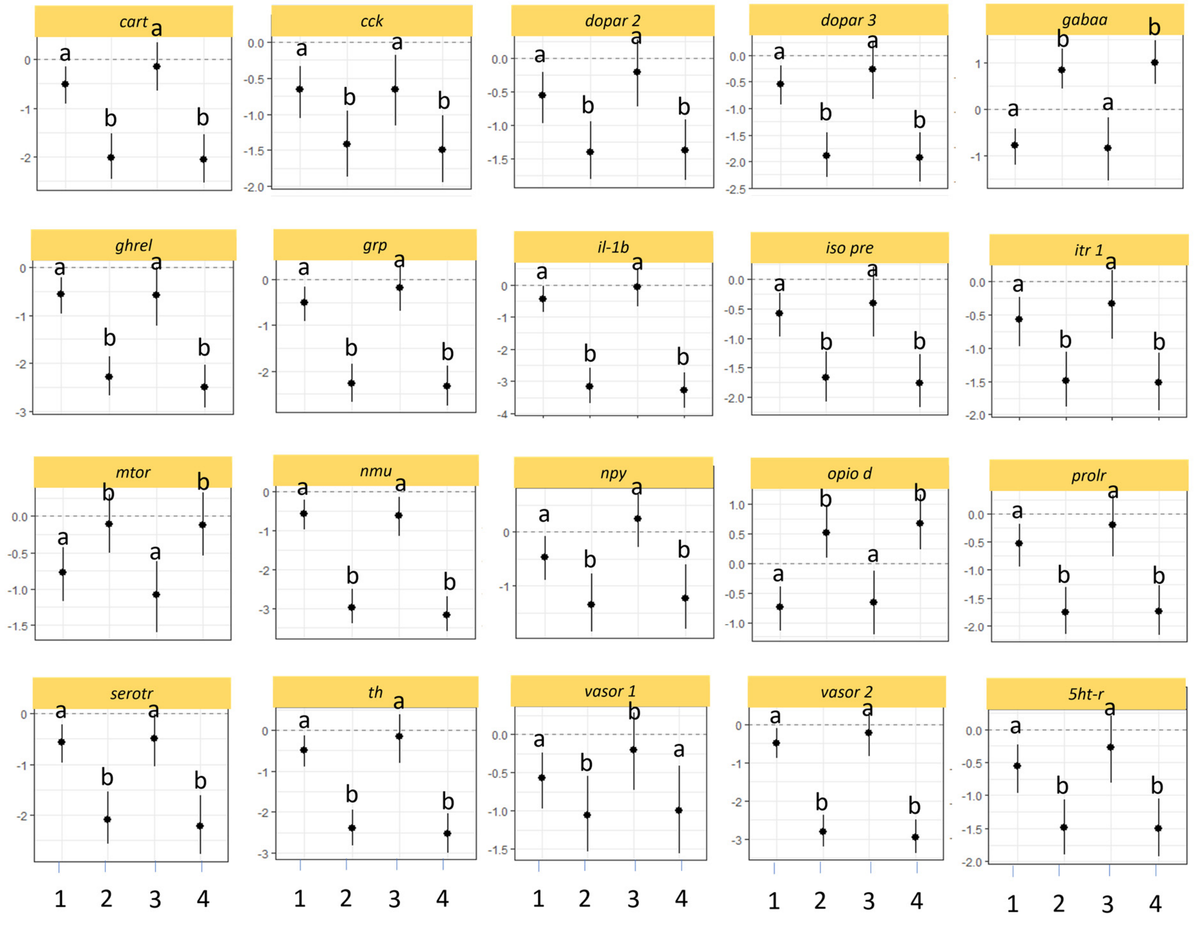

4E : Air

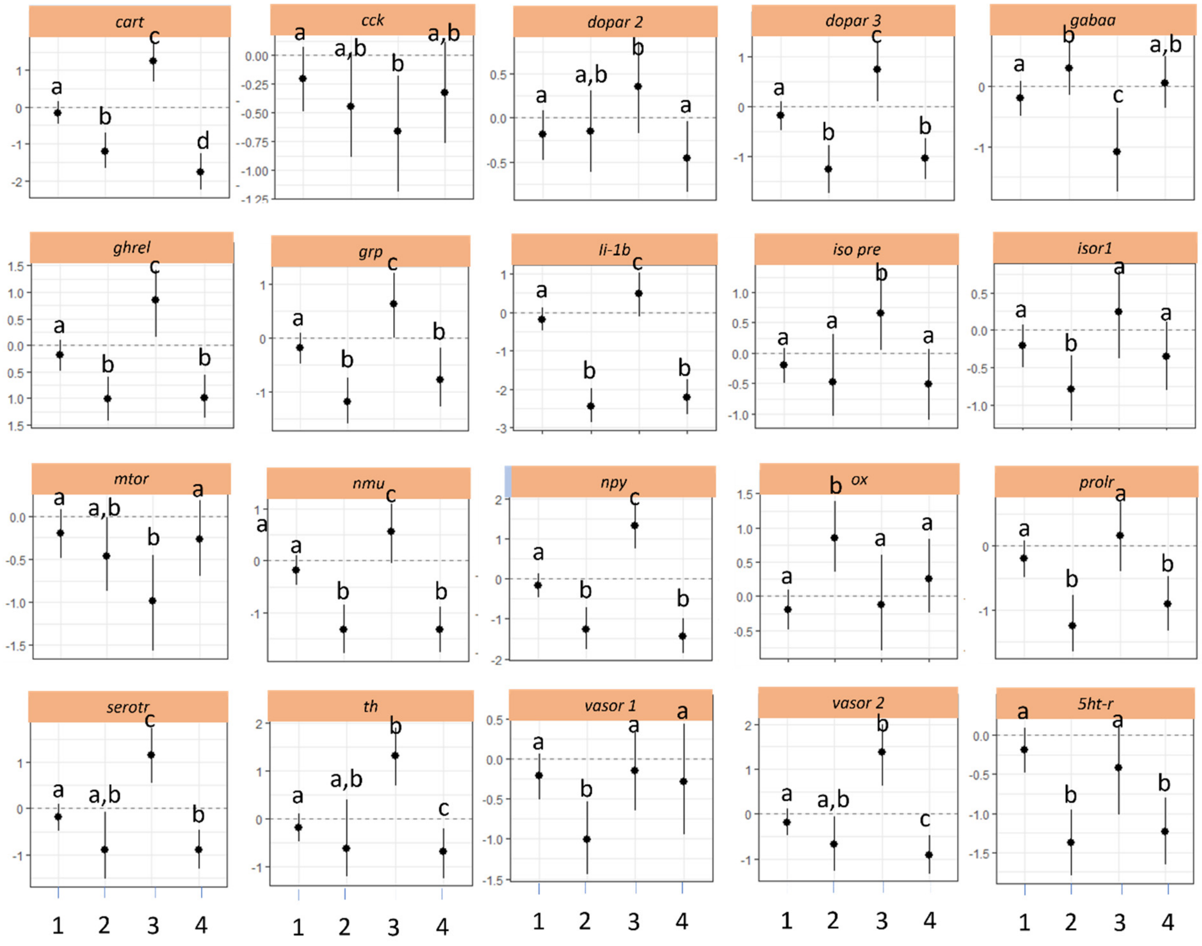

Figure S4. Gene expression profile in the rhombencephalon of control fish (group 1) and fish after the treatment (30 min = 2; 60 min = 3; 90 min = 4) whereby the following different treatments have been applied as described in the Material & Methods section: feed rewarding, feed control, chasing, confinement and air exposure, marginal means ± SEM; n = 6 per treatment, means of groups with the same letters are not significantly different from each other, $p > 0.05$).

In the rhombencephalon of carp, the expression of *npy* in feed-rewarded fish was significantly lower at 30 min after the treatment compared with controls and at 90 min after the treatment ($p \leq 0.0428$ Fig. S4A), whereas the expression of *ox* was only lower in the 30 min group than in the controls ($p = 0.010$). The expression of *ghrel* was lower 30 min and 60 min after feed rewarding compared with the controls ($p \leq 0.046$). In the rhombencephalon of carp, the expression of *mtor* in feed-rewarded fish was significantly higher and the expression of *th* lower in fish sampled 30 min after treatment compared with the controls and fish sampled after 90 min ($p \leq 0.044$; Fig. S4A). In the same fish, the expression of *vasor1* was lower in fish sampled 60 min after treatment compared with the controls ($p = 0.024$). The expression of *opiod* was higher and the expression of *il-1b* lower in fish sampled after 30 min and 60 min after treatment compared with the controls ($p \leq 0.030$). The feed control fish showed lower expression of *nmu* in the rhombencephalon at 60 min and 90 min after treatment compared with the controls and the 30 min group ($p \leq 0.042$; Fig. S4B). Quite similarly, the *ghrel* expression was low in the 60 min and 90 min groups compared with the controls ($p \leq 0.046$). The same fish showed higher expression of *cart* in the fish 30 min after treatment compared with the controls ($p = 0.002$). The expression of *npy* and *grp* was higher 30 min after feed control treatment than 60 min and 90 min after treatment ($p \leq 0.034$). The expression of *ox* was only lower 90 min after treatment compared with the controls ($p \leq 0.036$). The feed control fish showed lower expression of *5ht-r* and *il-1b* in the rhombencephalon of fish sampled 60 min and 90 min after treatment compared with the controls ($p < 0.038$; Fig. S4B). In addition, fish 30 min and 60 min after feed control treatment, higher *serotr*

expression was observed than in the controls ($p < 0.034$). Moreover, feed control fish showed higher *mtor* expression 90 min after treatment than in the remaining treatments ($p \leq 0.004$), and higher *gabaa* expression in fish sampled 60 min and 90 min after treatment compared with the controls and fish of the 30 min group ($p \leq 0.032$). The expression of *vasor 1* was lower in fish sampled after 60 min than in the controls ($p = 0.012$), and the expression of *th* and *vasor 2* lower 90 min after treatment than in the remaining treatment groups (≤ 0.002). The expression of *opiod* was higher 60 min after feed control treatment compared with the 30 min group and lower than in the 90 min group ($p \leq 0.008$). The rhombencephalon of feed control fish was also the only brain part that showed lower *prolr* expression 90 min after treatment compared with the fish sampled after 30 min ($p = 0.046$). In chased animals, the expression of *nmu* and *npy* in the rhombencephalon was lower in fish 30 min and 90 min after the treatment compared with the controls ($p \leq 0.038$; Fig. S4C). Moreover, all chased animals showed a lower expression of *ghrel* and *ox* in the rhombencephalon compared with the controls ($p \leq 0.038$). In chased animals, a lower expression of *5ht-r* was observed in the rhombencephalon in fish of the 60 min group compared with the controls ($p = 0.018$; Fig. S4C), whereas the expression of *serotr* was higher 90 min after treatment compared with the controls and fish sampled after 30 min ($p \leq 0.044$). In the same animals, the expression of the *mtor* and *gabaa* was higher in fish sampled 90 min after treatment compared with the controls (p ≤ 0.0-10). The expression of the *th* and *vasor 2* was lower and the expression of *opiod* higher in fish sampled 30 min and 90 min after treatment compared with the controls ($p \leq 0.034$). In addition, all chased fish showed a lower *il-1b* expression compared with the controls ($p \leq 0.010$). Confined fish showed a lower *nmu, npy, ox* and *cart* expression in fish sampled 30 min and 90 min after treatment compared with the controls and fish sampled after 60 min ($p \leq 0.018$; Fig. S4D). The confined fish also showed higher *grp* and *ghrel* expression in fish 30 min and 90 min after treatment, but higher *grp* expression in the 60 min group than in the controls ($p \leq 0.034$). Confined fish showed a lower *5ht-r, vasor 1, vasor 2, th, iso pre, il-1b* and *serotr* expression in fish of the 30 min and 90 min group compared with the other two groups ($p \leq 0.024$; Fig. S4D), but only the expression of *serotr* was higher in the 60 min group. By contrast, these fish showed a higher *mtor* expression 30 min and 90 min after treatment and a lower expression 60 min after treatment compared with the controls ($p \leq 0.001$). The higher expression at the 30 min and 90 min time points was also observed for the *gabaa* and the *opiod* expression in confined fish ($p \leq 0.028$). The expression of *prolr* was higher only at 60 min after treatment compared with the remaining groups ($p \leq 0.042$). Air-exposed fish showed a lower *nmu, npy, grp, ghrel* and *cart* expression in fish 30 min and 90 min after treatment and higher expression in the 60 min group compared with the controls ($p < 0.014$; Fig. S4E). In the same fish, a lower *cck* expression in fish 60 min after air exposure was observed compared with the controls ($p \leq 0.032$). Finally, air-exposed fish 30 min after treatment showed higher *ox* expression than the remaining groups ($p \leq 0.028$). Air-exposed fish showed a lower *5ht-r, dopar 3,* $il\text{-}1\beta$ and *prolr* expression in fish of the 30 min and 90 min groups compared with the controls and fish sampled 60 min after treatment ($p \leq 0.028$; Fig. S4E). The expression of *serotr* was higher between fish sampled 60 min after air exposure and lower in the 90 min group than in the controls ($p \leq 0.002$). In addition, the expression of *iso pre* was significantly higher at 60 min after treatment compared the remaining treatments ($p \leq 0.016$). Furthermore, the expression of *mtor* was lower and the expression of *dopar 2, vasor 2* and *th* higher in the 60 min group than in the controls and fish sampled after 90 min ($p \leq 0.038$). However, the expression of *gabaa* was higher in the 30 min group and lower 60 min after treatment compared with the controls ($p \leq 0.038$). The expression of *vasor 1* and *it-r1* was only lower in the 30 min group com-pared with the remaining groups ($p \leq 0.046$).

Supplementary Figure S5. Gene expression analysis with Principal Component Analyses (PCA) for the most contributing genes in the telencephalon showing their representation on the factor map as cos2 values for the 5 most contributing dimensions of the PCA of fish 0, 30 min, 60 min and 90 min after treatment; n = 6 per treatment.

Feed reward

Appetite

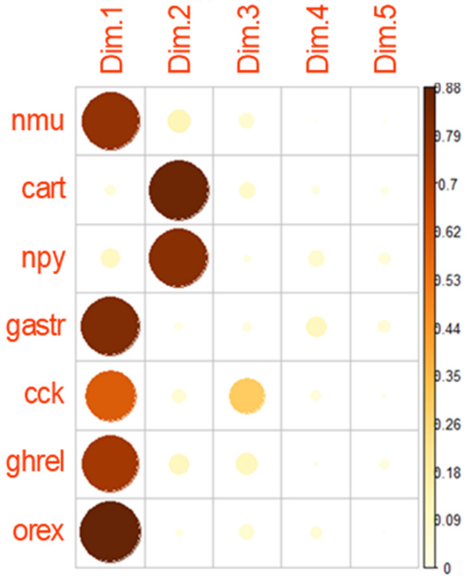

Other pathways

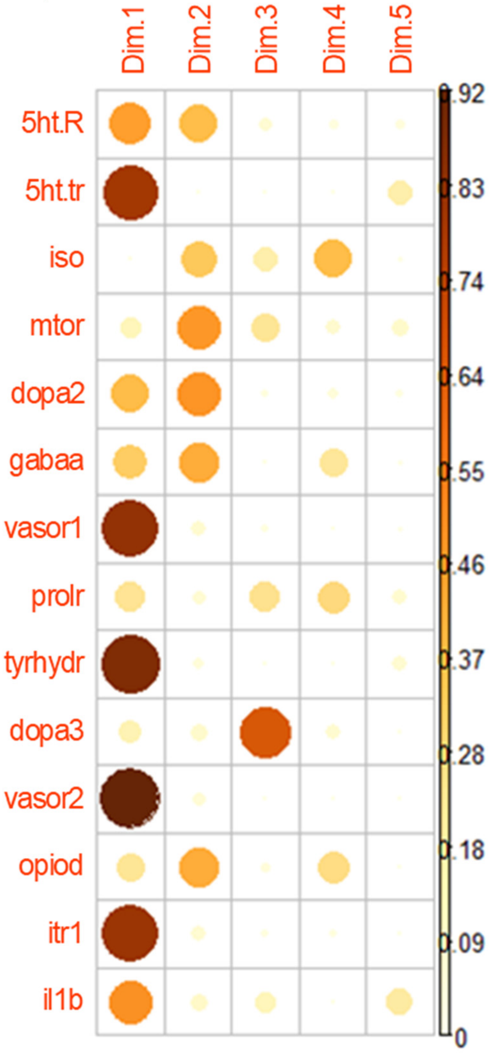

Feed control

Appetite

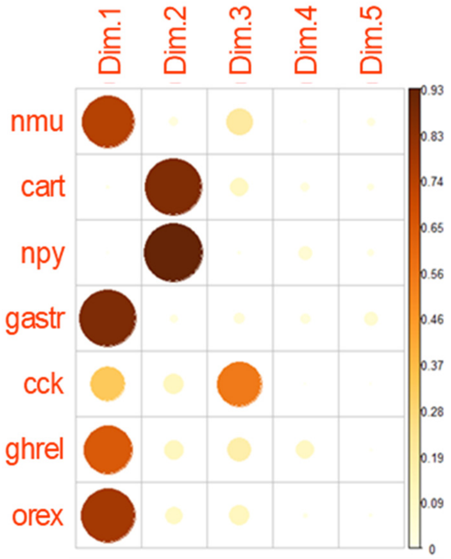

Other pathways

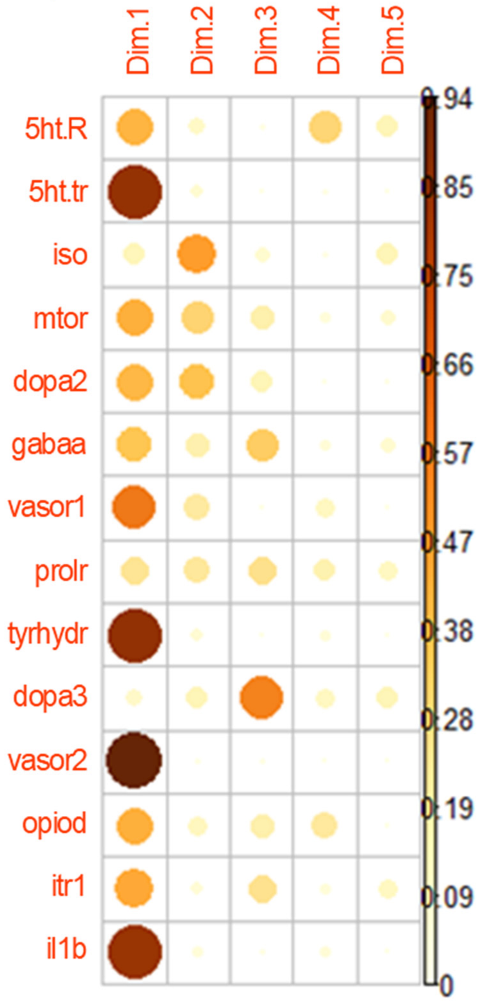

Chasing

Appetite

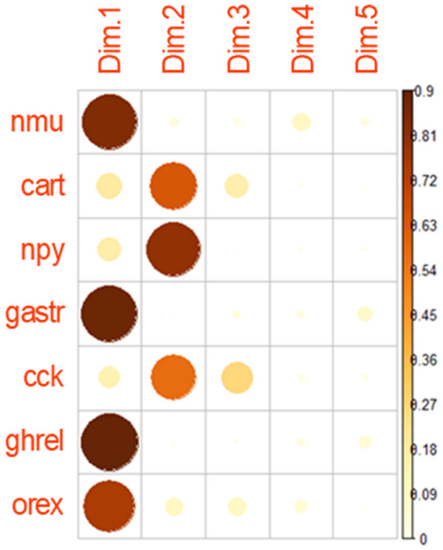

Other pathways

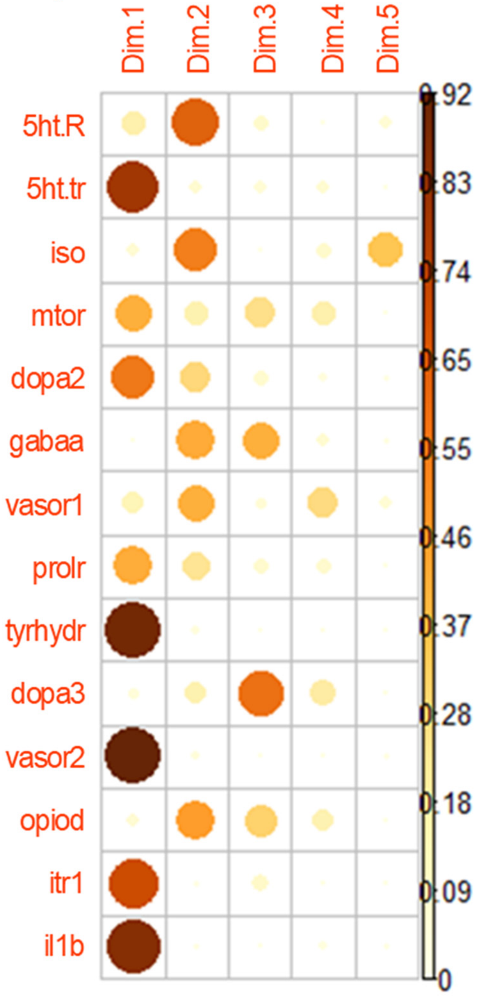

Confinement

Appetite

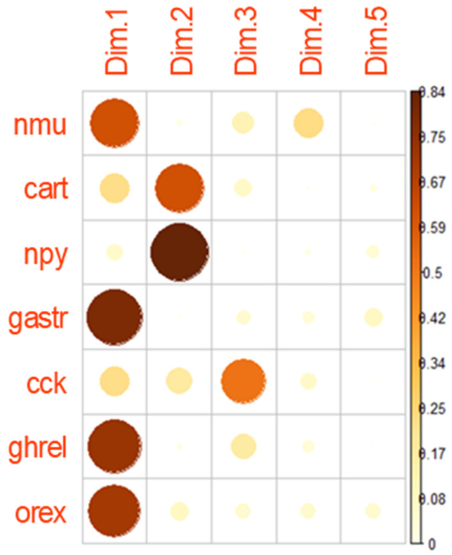

Other pathways

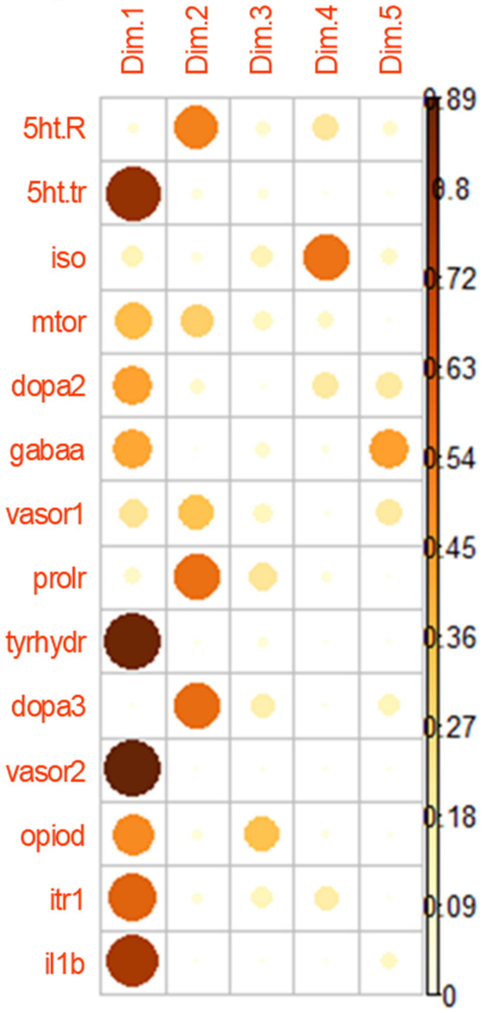

Air exposure

Appetite

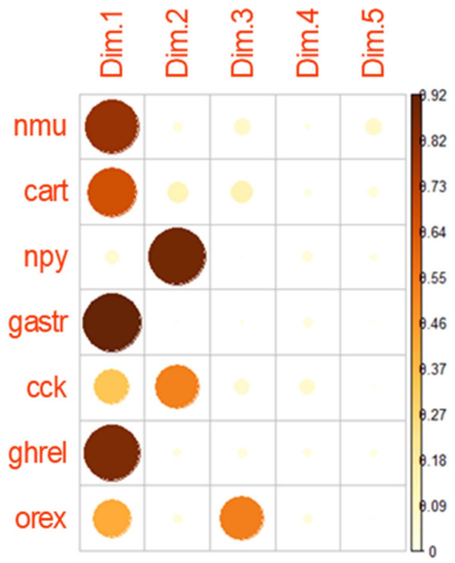

Other pathways

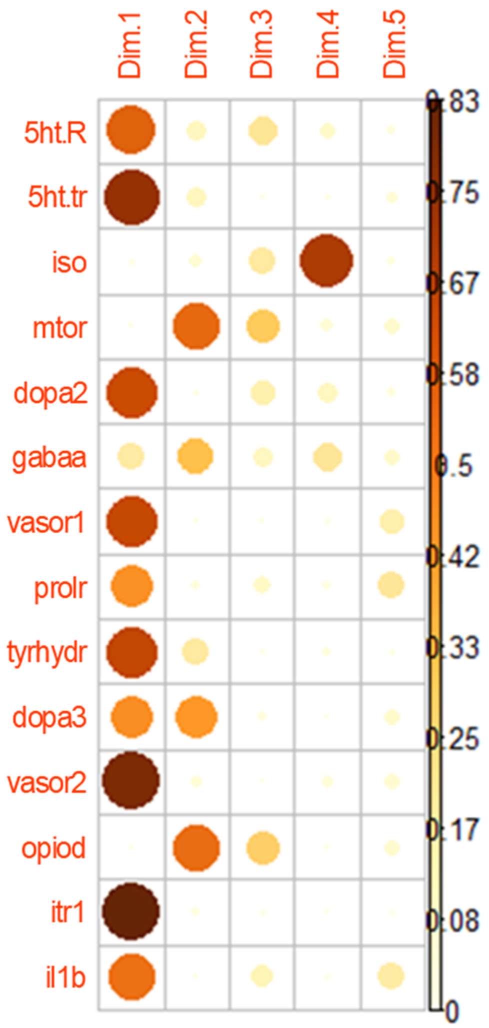

Supplementary Figure S6. Gene expression analysis with Principal Component Analyses (PCA) for the most contributing genes in the hypothalamus showing their representation on the factor map as cos2 values for the 5 most contributing dimensions of the PCA of fish 0, 30 min, 60 min and 90 min after treatment; n = 6 per treatment.

Feed reward

Appetite

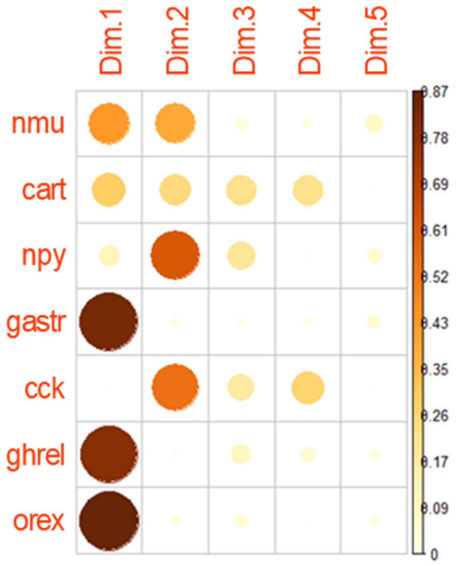

Other pathways

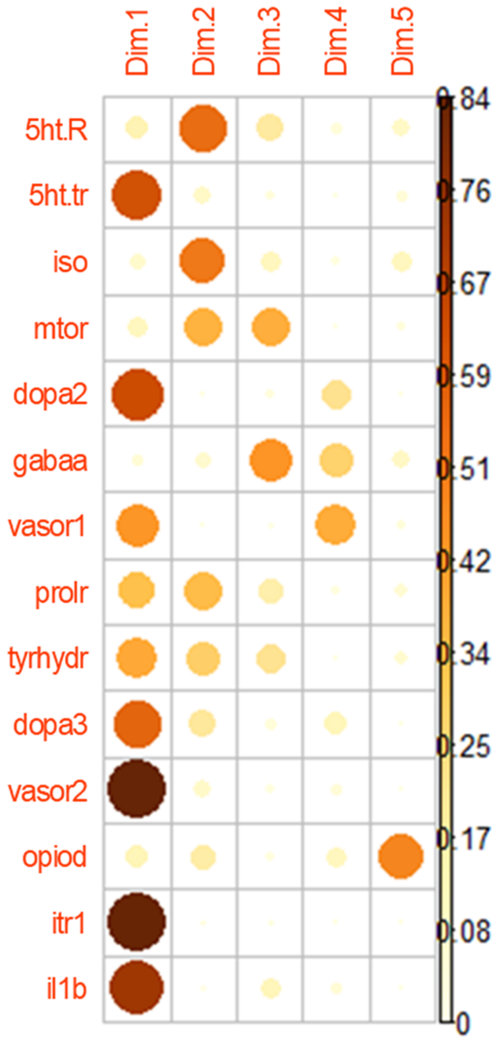

Feed control

Appetite

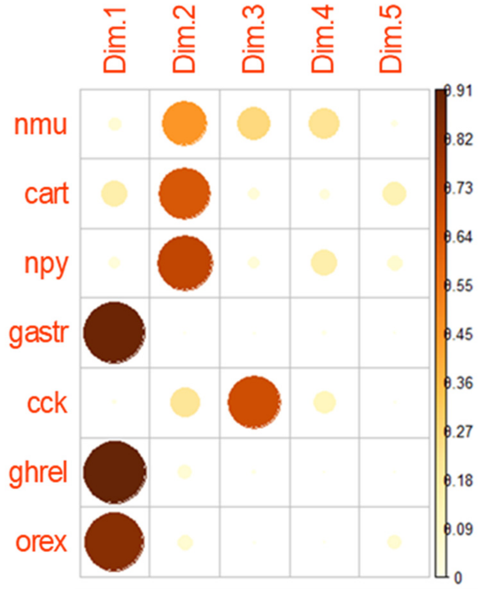

Other pathways

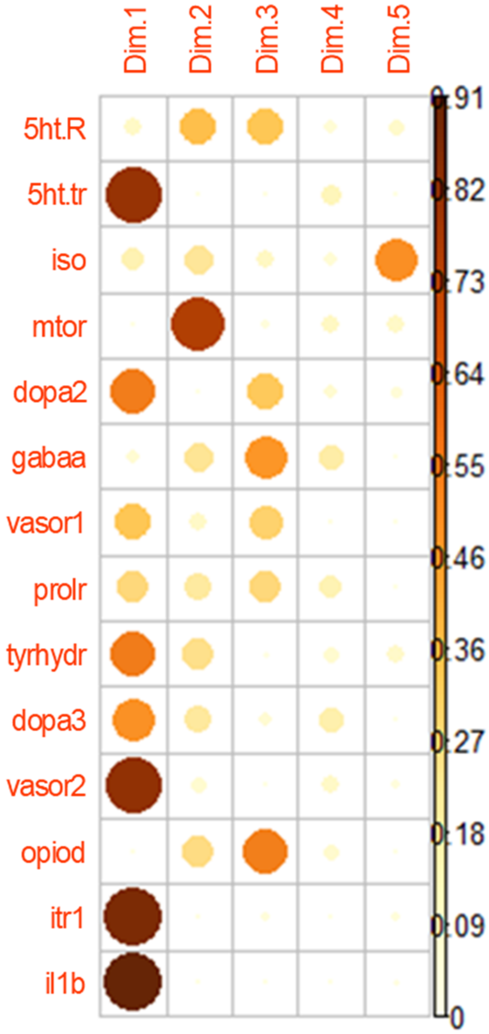

Chasing

Appetite

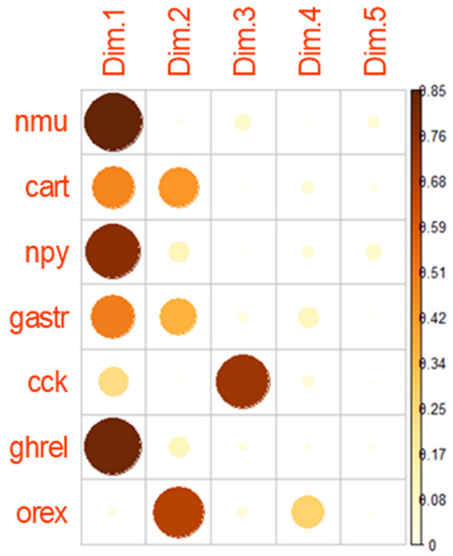

Other pathways

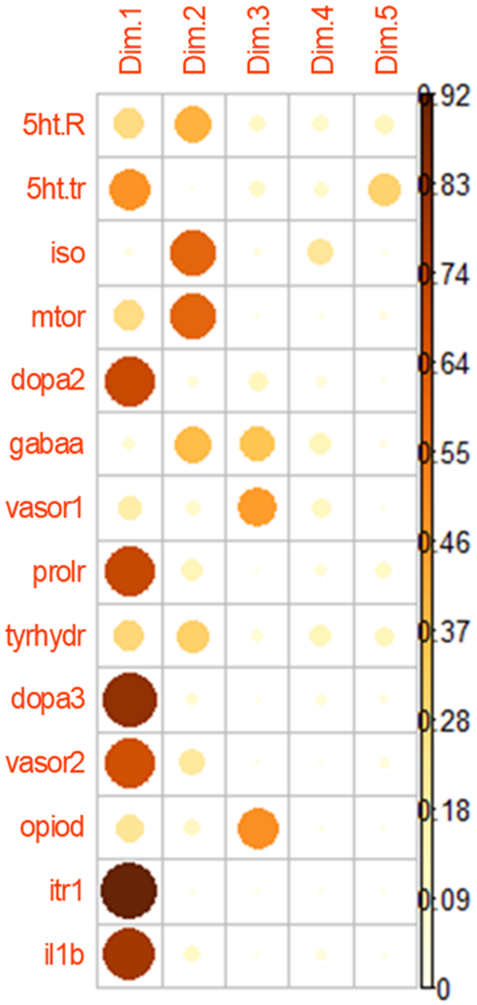

Confinement

Appetite

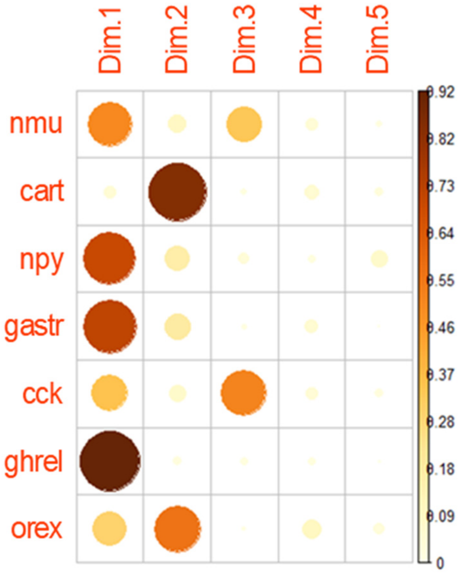

Other pathways

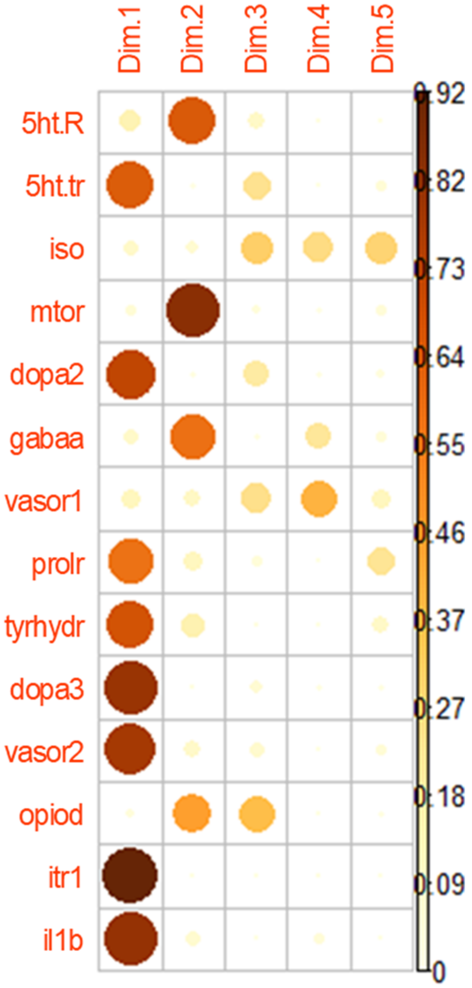

Air exposure

Appetite

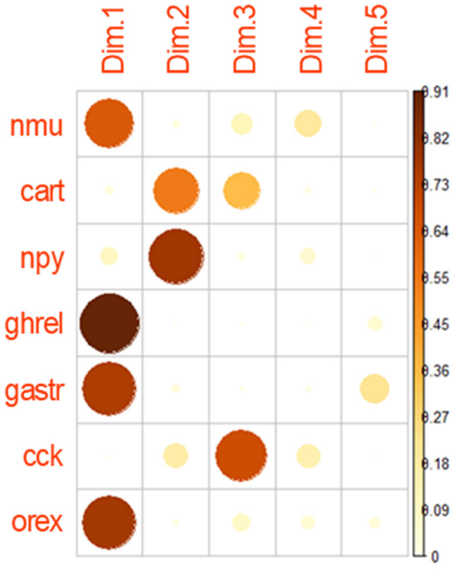

Other pathways

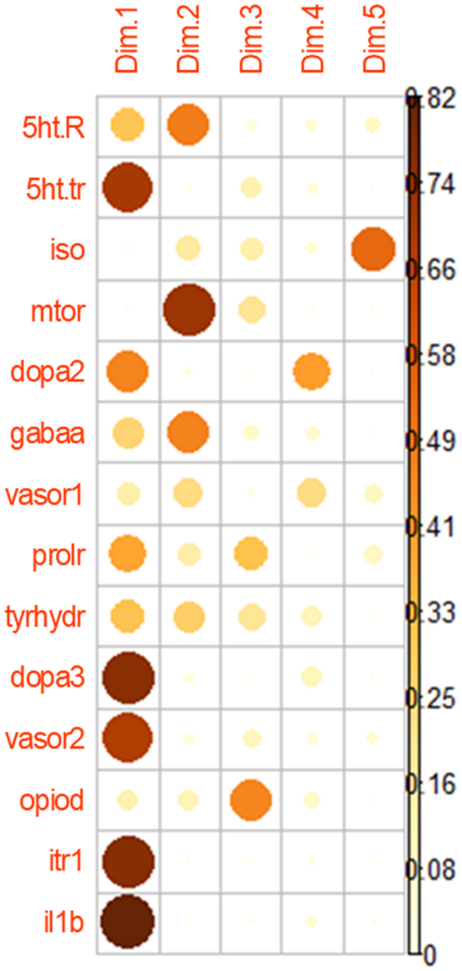

Supplementary Figure S7. Gene expression analysis with Principal Component Analyses (PCA) for the most contributing genes in the optic tectum showing their representation on the factor map as cos2 values for the 5 most contributing dimensions of the PCA of fish 0, 30 min, 60 min and 90 min after treatment; n = 6 per treatment.

Feed reward

Appetite

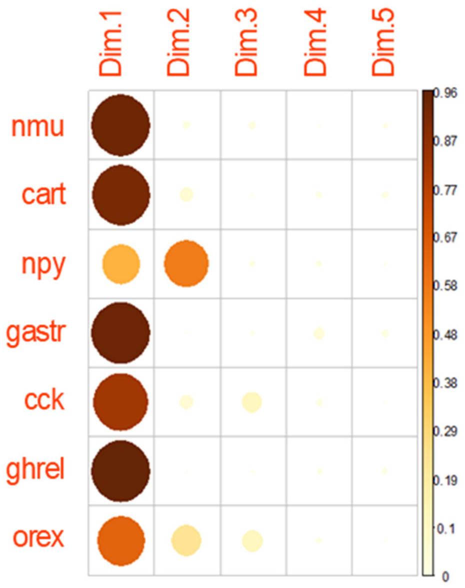

Other pathways

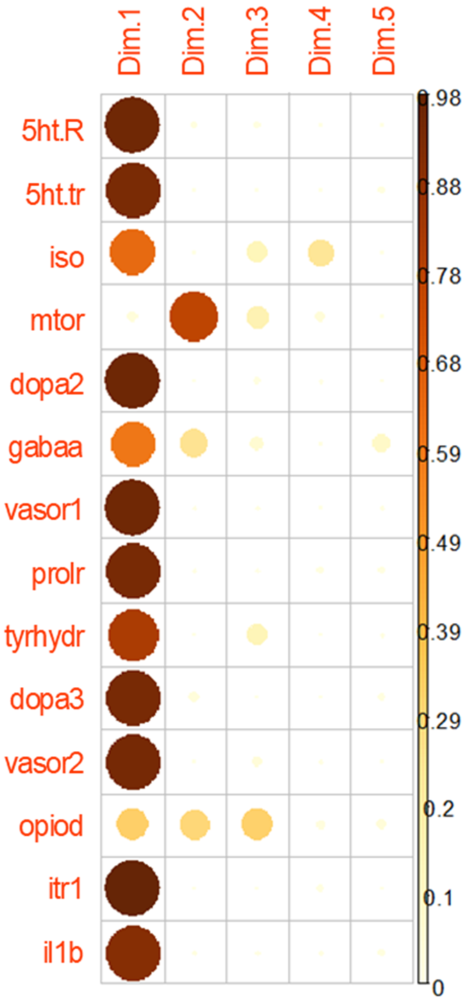

Feed control

Appetite

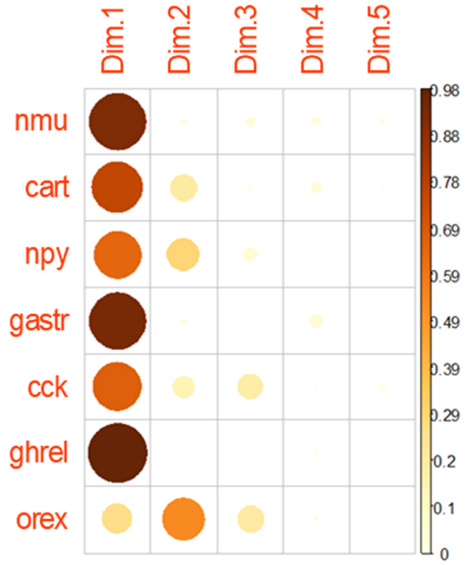

Other pathways

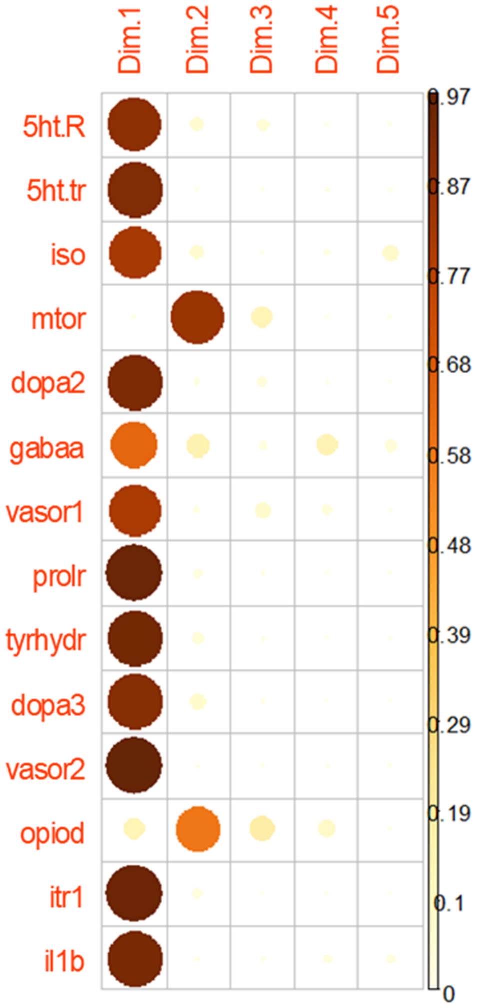

Chasing

Appetite

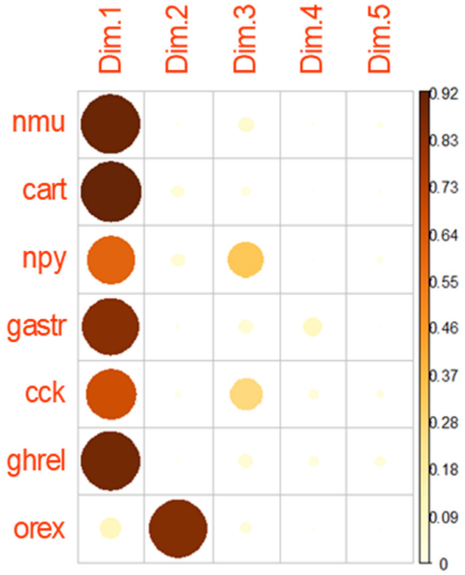

Other pathways

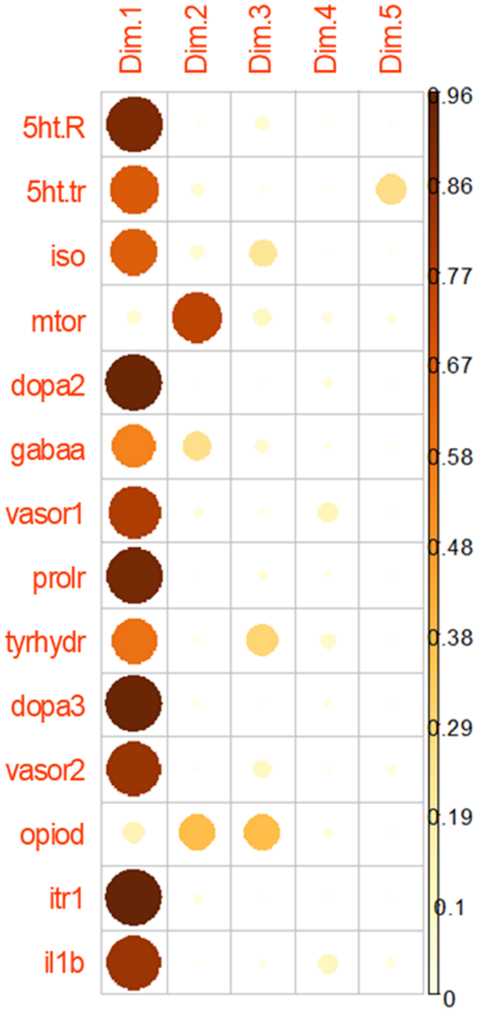

Confinement

Appetite

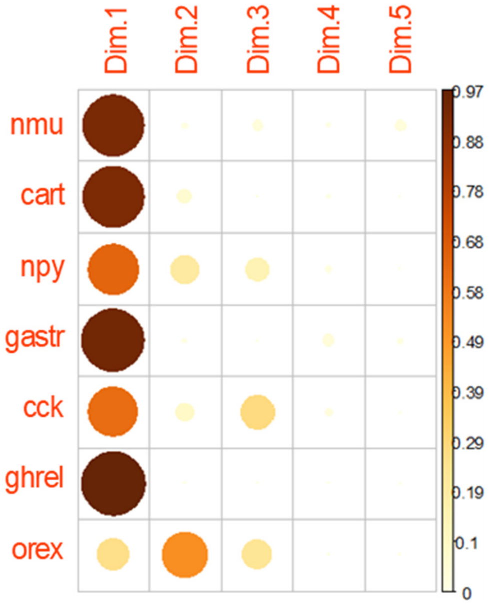

Other pathways

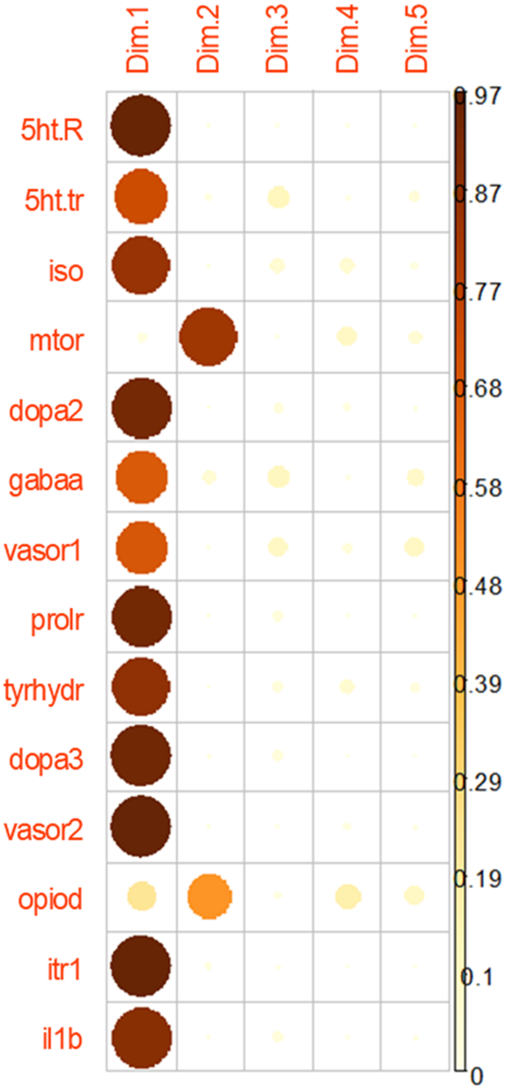

Air exposure

Appetite

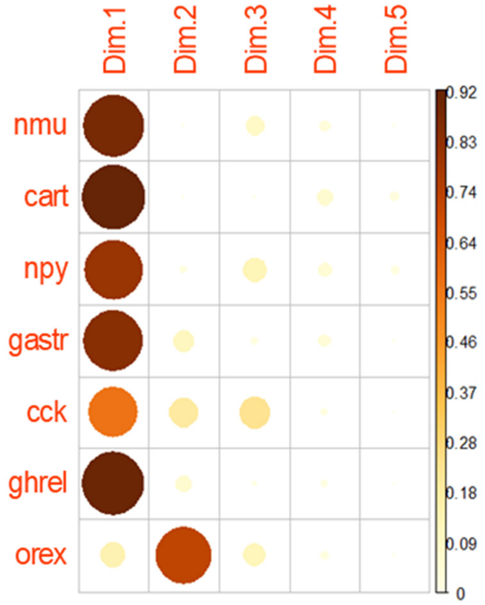

Other pathways

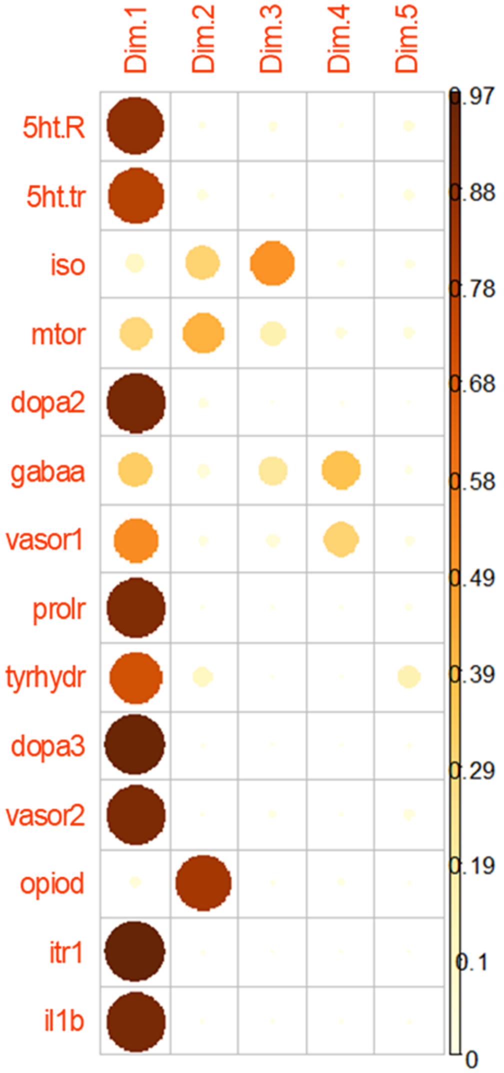

Supplementary Figure S8. Gene expression analysis with Principal Component Analyses (PCA) for the most contributing genes in the rhombencephalon showing their representation on the factor map as cos2 values for the 5 most contributing dimensions of the PCA of fish 0, 30 min, 60 min and 90 min after treatment; n = 6 per treatment.

Feed reward

Appetite

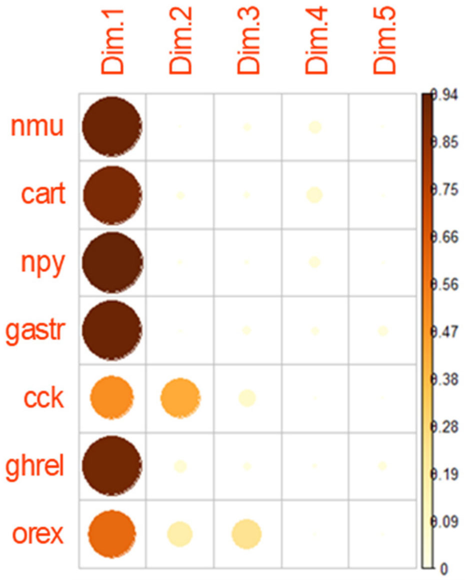

Other pathways

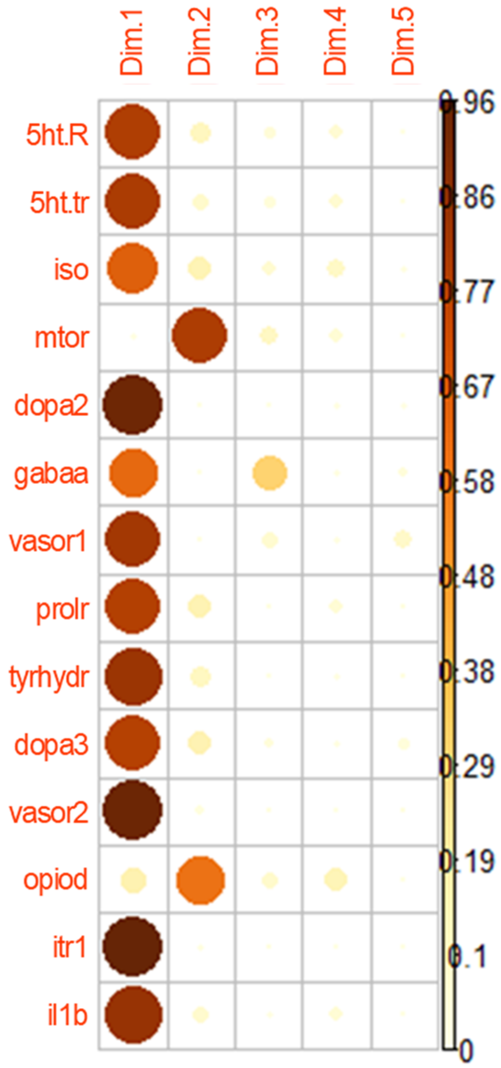

Feed control

Appetite

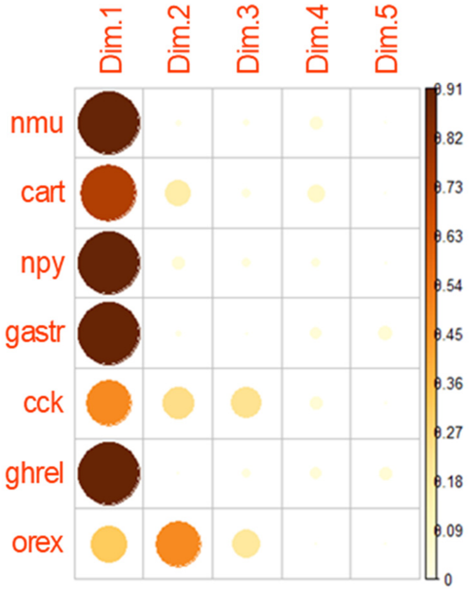

Other pathways

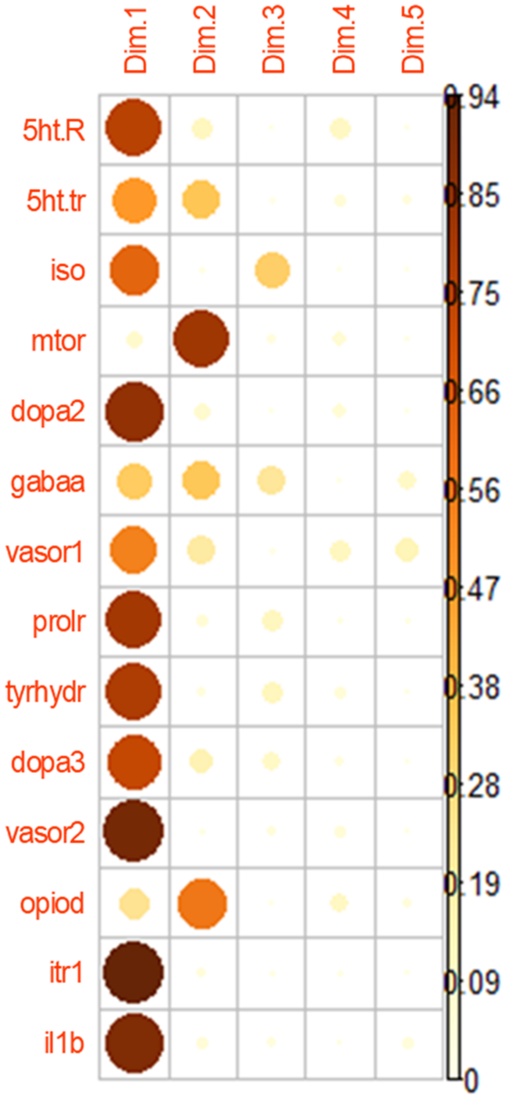

Chasing

Appetite

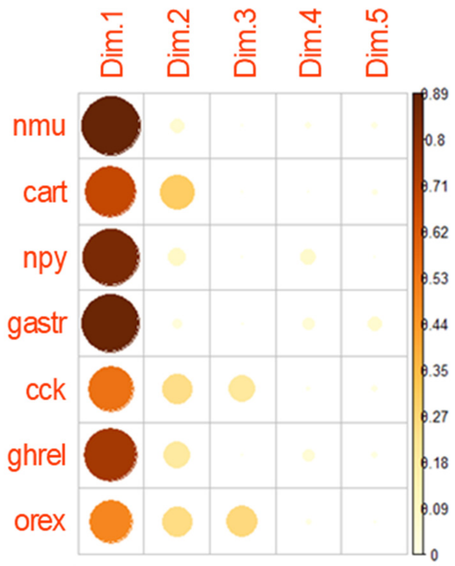

Other pathways

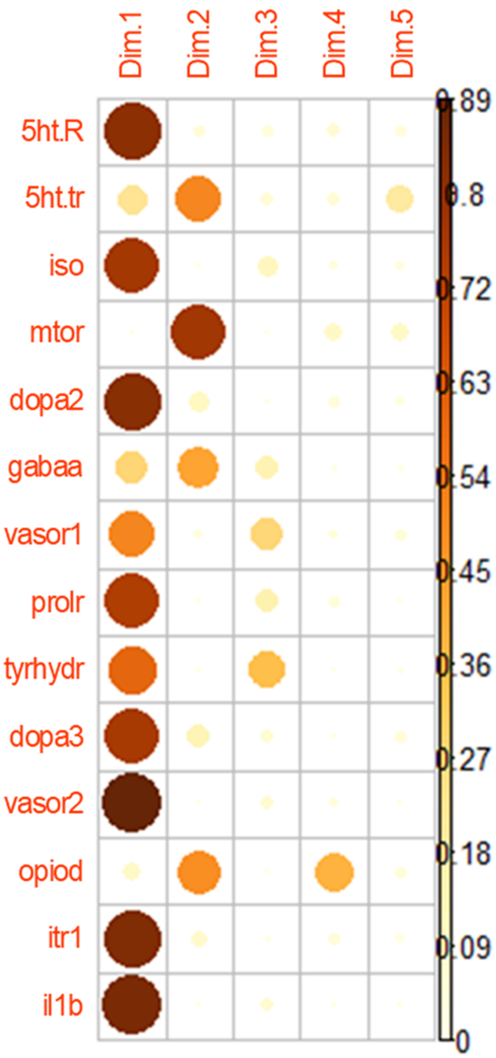

Confinement

Appetite

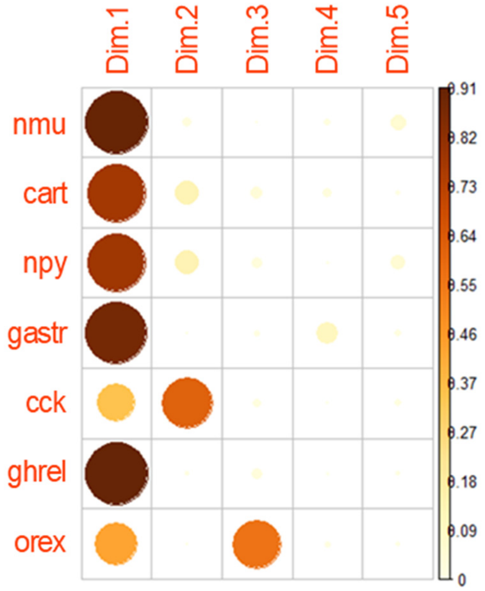

Other pathways

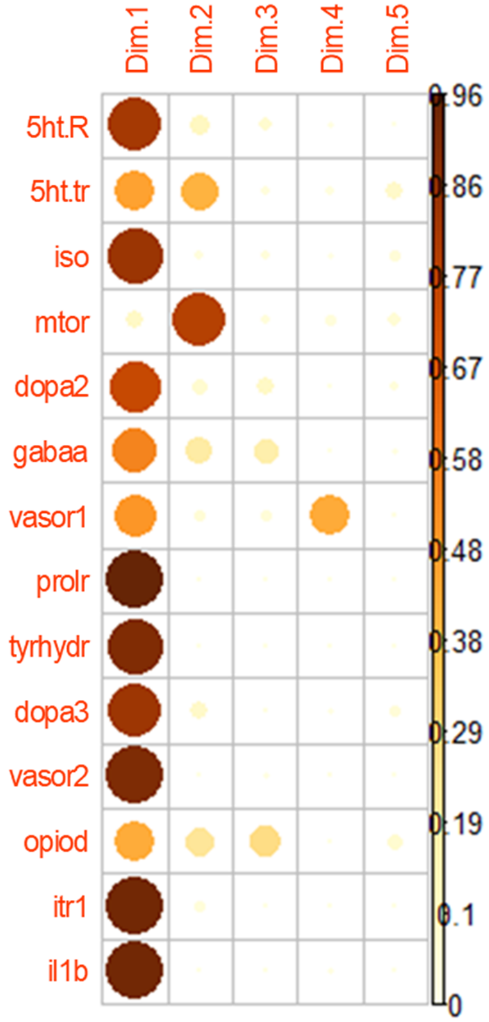

Air exposure

Appetite

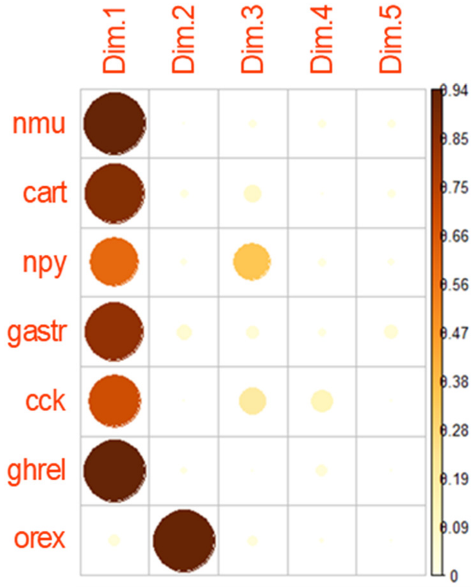

Other pathways

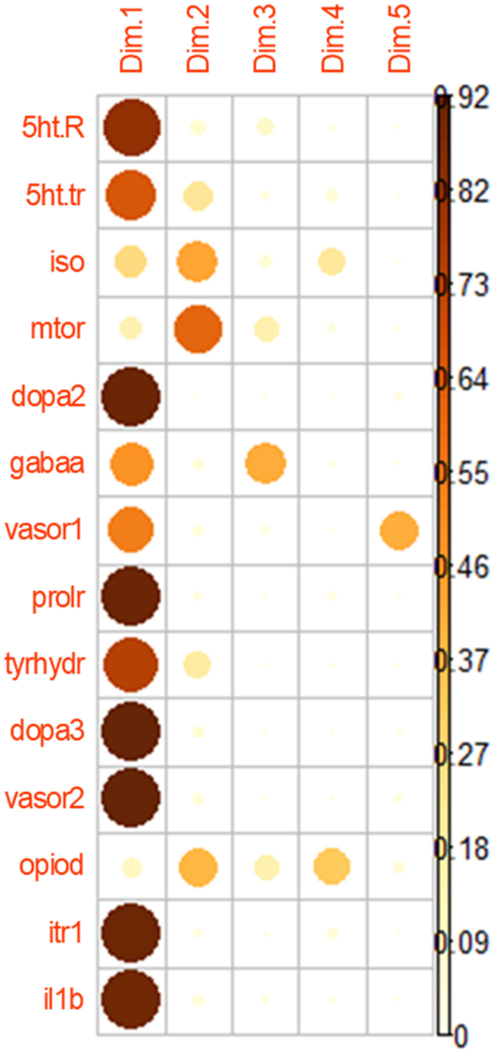

Supplement: Supplementary file 1 [file animals-14-03413-s001.zip › animals-3317833-supplementary.pdf]
